# A New Lyngbyatoxin from the Hawaiian Cyanobacterium *Moorea producens*

**DOI:** 10.3390/md12052748

**Published:** 2014-05-12

**Authors:** Weina Jiang, Wei Zhou, Hajime Uchida, Masayuki Kikumori, Kazuhiro Irie, Ryuichi Watanabe, Toshiyuki Suzuki, Bryan Sakamoto, Michiya Kamio, Hiroshi Nagai

**Affiliations:** 1 Department of Ocean Sciences, Tokyo University of Marine Science and Technology, Tokyo 108-8477, Japan; E-Mails: jwnkouina@gmail.com (W.J.); vivitalot@gmail.com (W.Z.); huchida.msr181823@gmail.com (H.U.); mkamio@kaiyodai.ac.jp (M.K.); 2 Division of Food Science and Biotechnology, Graduate School of Agriculture, Kyoto University, Kyoto 606-8502, Japan; E-Mails: kikumori@kais.kyoto-u.ac.jp (M.K.); irie@kais.kyoto-u.ac.jp (K.I.); 3 National Research Institute of Fisheries Science, Yokohama 236-8648, Japan; E-Mails: rwatanabe@affrc.go.jp (R.W.); tsuzuki@affrc.go.jp (T.S.); 4 Richard L. Roudebush VA Medical Center, Indianapolis, IN 46202, USA; E-Mail: bryan.sakamoto@va.gov

**Keywords:** lyngbyatoxin A, cyanobacteria, *Moorea producens*, toxicity, protein kinase C

## Abstract

Lyngbyatoxin A from the marine cyanobacterium *Moorea producens* (formerly *Lyngbya majuscula*) is known as the causative agent of “swimmer’s itch” with its highly inflammatory effect. A new toxic compound was isolated along with lyngbyatoxin A from an ethyl acetate extract of *M. producens* collected from Hawaii. Analyses of HR-ESI-MS and NMR spectroscopies revealed the isolated compound had the same planar structure with that of lyngbyatoxin A. The results of optical rotation and CD spectra indicated that the compound was a new lyngbyatoxin A derivative, 12-*epi*-lyngbyatoxin A (**1**). While 12-*epi*-lyngbyatoxin A showed comparable toxicities with lyngbyatoxin A in cytotoxicity and crustacean lethality tests, it showed more than 100 times lower affinity for protein kinase Cδ (PKCδ) using the PKCδ-C1B peptide when compared to lyngbyatoxin A.

## 1. Introduction

**Figure 1:**
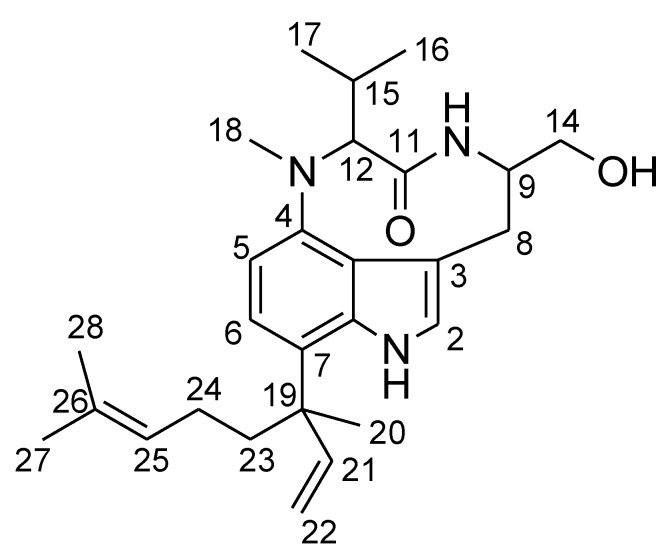
Structures of compounds **1**–**5**.

*Moorea producens* (formerly classified as *Lyngbya majuscula*) [1] a filamentous marine cyanobacterium can cause human skin irritation (seaweed dermatitis) known as “swimmer’s itch” [2]. The causative agents of “swimmer’s itch” have been reported to be lyngbyatoxin A, aplysiatoxin and their derivatives, which are produced by *M. producens* [3,4,5]. These toxins have been shown to possess potent tumor-promoting activity and ability to activate protein kinase C isozymes [6,7]. Fatal intoxication due to ingestion of lyngbyatoxin A contaminated flesh of the turtle *Chelonia mydas* has been reported [8,9]. In addition, aplysiatoxin and related toxins were revealed to be the causative agents of food poisoning by the red alga *Gracilaria coronopifolia* [10,11,12]. The true producer of these toxins involved in these poisoning cases was deduced to be *M. producens* [8,10]. Since these toxins produced by *M. producens* are suspected as fatal tumor-causing factors for marine animals, such as the green turtle and manatee [13,14], the study of toxins produced by *M. producens* is important from an ecotoxicological point of view. Additionally, *M. producens* is a rich source of unique compounds which has led to the extensive study of its bioactive compounds, that may lead to the discovery of novel therapeutics agents [15,16,17]. We examined the toxic components in the extracts of *M. producens* collected from Hawaii guided by the lethal activity toward crustaceans. A new lyngbyatoxin derivative (**1**, 12-*epi*-lyngbyatoxin A, Figure 1) was isolated along with lyngbyatoxin A (**2**, Figure 1). In this report, the isolation, structure and toxicities of compound **1** will be discussed.

**1**: 12-*Epi*-lyngbyatoxin A (9*S*,12*R*,19*R*); **2**: Lyngbyatoxin A (teleocidin A-1) (9*S*,12*S*,19*R*);

**3**: Teleocidin A-2 (9*S*,12*S*,19*S*); **4**: Enantiomer of **2 **(9*R*,12*R*,19*S*); **5**: Enantiomer of **3 **(9*R*,12*R*,19*R*).

## 2. Results and Discussion

Compounds **1** and **2** were both isolated as pale yellow gummy solids. Compound **2** was identified to be lyngbyatoxin A which had been first isolated from the Hawaiian cyanobacterium [3]. HR-ESI-MS of compound **1** exhibited a molecular ion peak at *m*/*z* 438.3070 [M + H]^+^, consistent with the molecular formula of C_27_H_39_N_3_O_2_, which was the same molecular formula with that of lyngbyatoxin A (**2**). The presence of an indole ring was suggested from its UV spectrum (λ_max_ (EtOH) nm (log ε) 231 (4.33), 301 (3.86)) comparing with that of **2**.

**Figure 2:**
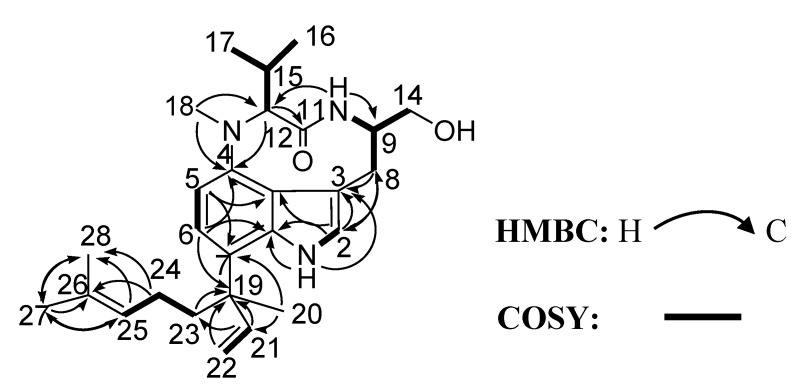
Key correlations of compound **1** in the COSY (bold line) and HMBC (arrow) spectra.

Comparison of the ^1^H and ^13^C NMR data of **1** with those of **2**, together with 2D NMR spectral analysis led us to elucidate the planar structure of the new compound as **1** (Figure 2). The planar structure of **1** was completely the same as that of lyngbyatoxin A (**2**). ^1^H and ^13^C NMR spectral data for **1** were shown in Table 1. On ^1^H NMR, most of the chemical shifts of **1** were closely similar to those of **2** (see Supplementary Information, Table S1). However, some proton chemical shifts (for example, H-9, H-12 and H-14) on a nine-membered lactam ring were somewhat different from those of **2**. From these observations, **1** was deduced to have the same planar structure with **2**. However, the absolute configuration around the nine-membered lactam ring appeared to be different between **1** and **2**.

**Figure 3:**
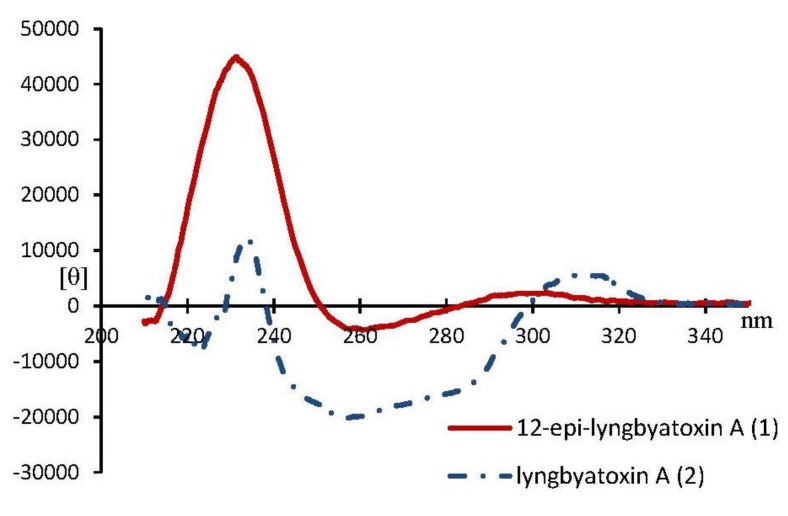
CD spectra of compounds **1** and **2**.

Lyngbyatoxin A (**2**) was first isolated as the causative agent of seaweed dermatitis from the cyanobacterium [3]. Later lyngbyatoxin A was found to be identical to teleocidin A-1 (**2**), the toxic metabolite from the actinomycete *Streptomyces mediocidicus* [18]. Teleocidin A-2 (**3**, Figure 1) of which C-19 had *S* configuration was also reported from *S. mediocidicus* [18]. The only structural difference between **2** and **3** was the configuration on C-19 in the linalyl group side chain. The circular dichroism (CD) spectra of compounds **2** and **3** showed only difference at around 230 nm (see Supplementary Information, Figure S22) [19,20]. The CD spectra around 230 nm of **2** and **3** showed upward and downward curves, respectively. Furthermore, CD spectra around 230 nm of **4** and **5** (synthesized compounds, Figure 1) showed downward and upward curves, respectively (see Supplementary Information, Figure S22) [20]. These results indicated the absolute configurations on C-19 of the linalyl group in lyngbyatoxin A derivatives were defined around 230 nm (CD spectra) as 19-*R* and 19-*S* configurations which resulted in upward and downward curves, respectively. CD spectra of **1** and **2** were shown in Figure 3. Both compounds showed upward curves around 230 nm, indicated that compound **1** had the same absolute configuration *R* at C-19 with that of **2**. In addition, the CD spectra of compounds **2** and **5** showed differences at around 220 nm and 270 nm. The spectra around 220 nm and 270 nm of compound **2** showed both downward curves, while compound **5** showed both upward curves. The same spectral tendencies were observed in compounds **3** and **4** (see Supplementary Information, Figure S22) [20]. The both upward curves at 220 and 270 nm meant C-9 (*R*) and C-12 (*R*), while both downward curves meant C-9 (*S*) and C-12 (*S*). On the other hand, the CD spectra of compound **1** showed upward curve at 220 nm and downward curve at 270 nm. The results indicated that compound **1** had 9*S*,12*R* or 9*R*,12*S* configurations. Taking this into consideration, it was supposed that the absolute configurations of compound **1** were 9*S*,12*R*,19*R* or 9*R*,12*S*,19*R*.

**Table 1:** NMR spectroscopic data for compound **1** in CDCl_3_.

No.	δ_C_^a^	δ_H_ (*J* in Hz) ^b^	HMBC
1		8.45, s	3, 3a, 7a
2	121.8	6.81, d (1.9)	3, 3a, 7a, 8
3	113.8		
3a	120.9		
4	146.7		
5	109.0	6.73, d (8.1)	3, 3a, 4, 7
6	119.8	6.95, d (8.1)	4, 5, 7a, 19
7	122.7		
7a	136.6		
8	32.3	3.27, dd (15.6, 2.5)	2, 3, 4, 9, 14
		2.90, dd (15.6, 2.5)	
9	57.6	3.84, br, m	
10		7.45, br, s	9, 12, 14
11	175.2		
12	69.0	3.91, d (10.6)	4, 11, 15, 17
14	65.4	3.88, dd (10.4, 3.6)	8, 9
		3.82, dd (10.4, 6.5)	
15	28.1	2.61, m	12, 16, 17
16	20.0	0.67, d (6.6)	12, 15, 17
17	20.4	0.74, d (6.5)	12, 15, 16
18	31.6	3.09, s	4, 12
19	43.4		
20	24.7	1.44, s	7, 19, 21, 23
21	148.8	6.21, dd (17.7, 10.6)	7, 19, 23
22	112.2	5.29, dd (17.9, 1.4)	19, 21
		5.26, dd (10.7, 1.4)	
23	38.2	1.98, m	7, 19, 21, 24, 25
		1.81, m	
24	22.7	1.91, br, m	23, 25, 26, 28
		1.71, br, m	
25	124.6	5.08, t (7.1)	23, 24, 27, 28
26	131.5		
27	17.4	1.40, s	25, 26, 28
28	25.7	1.64, s	25, 26, 27
OH on 14		Not observed	

^a^ Recorded at 200 MHz; ^b^ Recorded at 800 MHz. Coupling constants (Hz) are in parentheses. Abbreviations: s, singlet; d, doublet; t, triplet; m, multiplet; br, broad.

**Figure 4:**
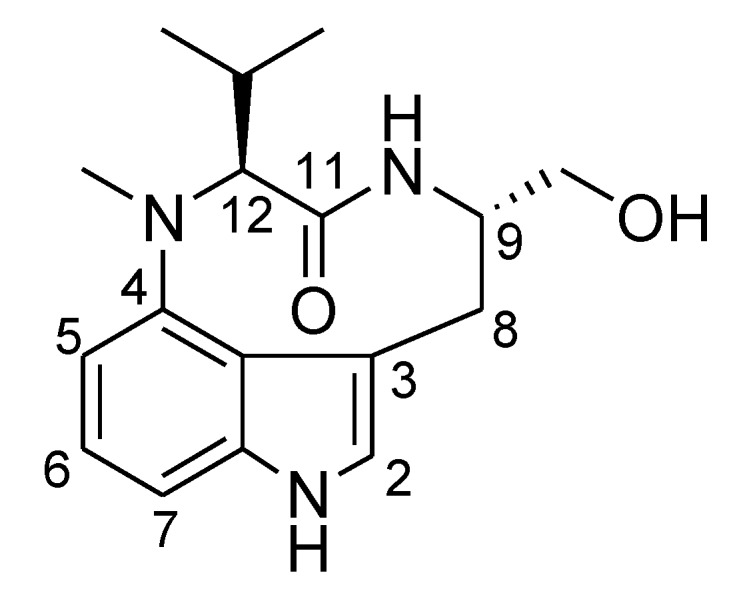
The structure of (−)-indolactam-V (**6**).

The ^1^H NMR spectrum of **2** in CDCl_3_ showed that it existed as a mixture of *trans* and *cis* amide conformers. The conformational ratio of lyngbyatoxin A was about 1:3 (*trans*:*cis*) (see Supplementary Information, Figure S24). This conformational ratio was almost the same as that of (−)-indolactam-V (IL-V, **6**, Figure 4) [21,22], which is regarded to be a core structure exhibiting tumor-promoting activity of teleocidins (lyngbyatoxins) [23]. IL-V (**6**), which has chiral carbon centers at C-9 and C-12 with the configurations of *S* and *S*, is a partial structure of **2**. Therefore, the enantiomer (9*R*,12*R*) of IL-V should also exist as two conformers of which the ratio is about 1:3 (*trans*:*cis*) in CDCl_3_. In this study, it was shown that compound **1** had nearly one conformer on the ^1^H NMR spectrum (see Supplementary Information, Figure S23). It was reported that (+)-*epi*-IL-V (9*S*,12*R*) should have more than two conformers by the computational calculations [24]. However, it had been shown that the main conformation was dominant for (+)-*epi*-IL-V (9*S*,12*R*) on the ^1^H NMR spectrum as in the case of compound **1** [21,24]. Therefore, the absolute configurations at C-9 and C-12 in the new lyngbyatoxin derivative (**1**) were inferred to be *S* and *R* or *R* and *S*, respectively, which further certified the conclusion drawing from the analyses of the CD spectra.

IL-V (**6**) and a number of related compounds have been isolated from natural samples or synthesized [3,18,19,20,25,26,27,28,29,30,31,32,33]. Those reports showed that all the reported compounds containing **6** (9*S*,12*S*) so far had levorotatory optical rotations no matter what the terpene group was connected to the indole ring (see Supplementary Information, Figures S26 and S27). However, we should pay attention that the levorotatory optical rotation of IL-V is determined not only by the 9*S*,12*S* configuration, but also by the main conformer of *cis* amide in the solution [30,34]. The synthetic IL-Vs (9*S*,12*S*) and (9*R*,12*S*) showed levorotatory optical rotation while IL-Vs (9*R*,12*R*) and (9*S*,12*R*) had dextrorotatory optical rotation (see Supplementary Information, Figures S26 and S27) [26,28]. Since the optical rotation of **1** was dextrorotatory, the configurations of indolactam (IL-V) of **1** were deduced to be 9*R*,12*R* or 9*S*,12*R*. Moreover, the absolute configurations at C-9 and C-12 in compound **1** were inferred to be 9*S*,12*R* or 9*R*,12*S* from the results of CD analysis. The absolute configuration of C-19 has been deduced as *R* also from CD spectra. When taken these results together, it was deduced that compound **1** had 9*S*,12*R*,19*R* absolute configurations. Furthermore, the deduced absolute chemistry of indolactam of **1** (9*S*,12*R*) was also supported by the ^1^H NMR spectra of **1** in CD_3_OD which was superimposable with those of (+)-*epi*-indolactam V (see Supplementary Information, Figure S25, Table S2) [28]. Thus, leading us to conclude that compound **1** was 12-*epi*-lyngbyatoxin A. This is the first report of 12-*epi*-lyngbyatoxin A (**1**) from the nature. Compound **1** had been obtained during the synthetic study of lyngbyatoxin A (**2**, teleocidin A-1) as one of the inseparable mixtures [20].

**6** (−)-indolactam-V.

In cytotoxic assays using L1210 leukemia cells, the IC_50_ values of **1** and **2** were 20.4 μM and 8.1 μM, respectively. The IC_50_ value of **1** was slightly higher than that of **2**. Both compounds showed moderate cytotoxicity. Compound **2** was formerly reported to exhibit cytotoxic effects against HeLa and ACC-MESO-1 cells with IC_50_ of 35 μM and 11 μM, respectively [32]. These values are comparable to the cytotoxic activities of **1** and **2** obtained in this study.

In the crustacean lethal activity tests using the shrimp *Palaemon paucidens*, both **1** and **2** showed similar lethal activities. The LD_100_ value of **1** was 7.5 mg/kg, while **2** was 5 mg/kg. Injection of **1** and **2** caused paralysis on the tested shrimp. Paralysis was not observed with compound **2** if the dose was less than the lethal amount. However, it was observed that compound **1** caused paralysis with a dose of 0.9 mg/kg. These results may indicate that **1** and **2** have different toxicity pathways.

Lyngbyatoxin A (**2**, teleocidion A-1) and related compounds are known as potent tumor promoters [6]. These compounds bind to the cysteine-rich C1 domains (C1A and C1B) of protein kinase C (PKC) isozymes to activate them, possibly leading to tumor formation. Affinities of the isolated compounds for PKCδ using a synthetic PKCδ-C1B peptide were evaluated by competition binding assay with [^3^H]phorbol 12,13-dibutyrate (PDBu). The values of *K_i_* for the inhibition of [^3^H]PDBu-binding were 17 nM and 0.11 nM for **1** and **2**, respectively. Aplysiatoxin (ATX) and debromoaplysiatoxin (DATX) are 12-*O*-tetradecanoylphorbol-13-acetate (TPA)-type tumor promoters which activate PKC as with lyngbyatoxin A (**2**) [6,29]. It was reported that *K_i_* values of ATX and DATX for binding to PKCδ-C1B are 0.41 nM [35,36] and 0.20 nM [37], respectively. These *K_i_* values are comparable to that of compound **2** obtained in this study. On the other hand, the affinity for PKCδ-C1B binding of compound **1** was more than a hundred times weaker than that of compound **2**. The results suggest the C12 configuration of the indolactam moiety of lyngbyatoxin A is very important for the binding with the PKCδ-C1B peptide.

Our results showed the *K_i_* value for PKCδ-C1B peptide binding of lyngbyatoxin A (**2**) was similar to those of other PKC activators aplysiatoxin and debromoaplysiatoxin. Lyngbyatoxin A (**2**) has been reported to be a potent tumor promoter and have the ability to strongly induce PKC activity [6,7,29]. While 12-*epi*-lyngbyatoxin A (**1**) and lyngbyatoxin A (**2**) showed comparable cytotoxic and crustacean lethal activities, 12-*epi*-lyngbyatoxin A (**1**) had more than 100 times lower binding affinity for PKC compared to lyngbyatoxin A (**2**). The results revealed that the cytotoxic and lethal mechanism of lyngbyatoxin-type compounds might be mediated through a non-PKC activation pathway. In this connection, there was a report about no correlation between the cytotoxicity and ability to bind to and activate PKCδ when examining the contribution of PKCδ to the cytotoxicity of aplysiatoxin related compounds [38]. Therefore, other targets that offer alternatives to PKC isozymes might exist for the expression of toxic activities by lyngbyatoxin-type compounds. Further study is necessary to verify this hypothesis.

In addition, lyngbyatoxin A (**2**) was reported as the causative agent of human skin irritation and marine turtle poisoning [3,8,9]. Since the new lyngbyatoxin derivative (**1**) has the similar cytotoxicity and lethal activity with lyngbyatoxin A (**2**), it is supposed that 12-*epi*-lyngbyatoxin A (**1**) is also a causative agent possibly.

## 3. Experimental Section

### 3.1. General Experimental Procedures

HPLC was performed using a SHIMADZU HPLC (SHIMADZU Co., Kyoto, Japan) pump equipped with a SPD-M10A diode array detector. HR-ESI-MS spectra data were determined using Bruker micrOTOF QII (Bruker Co., Bremen, Germany) mass spectrometer. NMR spectra were recorded in CDCl_3_ at 800 MHz and 200 MHz for ^1^H and ^13^C on a Bruker AVANCE III 800 MHz (Bruker Co., Bremen, Germany) spectrometer or in CD_3_OD at 600 MHz for ^1^H on a Bruker AVANCE III 600 MHz spectrometer; the chemical shifts were reported in δ units (ppm) using CDCl_3_ solvent (δ_H_ at 7.3 ppm and δ_C_ at 77.2 ppm) or using CD_3_OD solvent (δ_H_ at 3.3 ppm) as the internal standard signals. Optical rotations were measured on a JASCO P-2100 series.CD spectra were measured on a JASCO J-715 (JASCO Co., Tokyo, Japan) and UV spectra were on a HITACHI U-3000 (Hitachi High-Tech Fielding Co., Tokyo, Japan) spectrometer.

### 3.2. Marine Cyanobacterium Moorea producens

Samples of a marine cyanobacterium, *Moorea producens*, were collected at Kahala Beach, Oahu island, Hawaii in 1998. After freeze-drying, samples were stored at −30 °C until experiments were performed.

### 3.3. Extraction and Isolation

The dried sample (823 g dry wt.) was extracted twice with ethanol, six times with methanol and five times with acetone (see Supplementary Information, Figure S28). After evaporation of the solvents, the extracts were combined and partitioned between hexane and 80% methanol. The 80% methanol layer was again evaporated and partitioned with a mixture of ethyl acetate (EtOAc) and distilled water. The fraction of distilled water was then dissolved with butanol and separated into two extracts. The ethyl acetate fraction was then subjected to an open glass column 20 × 300 mm packed with ODS resin (Pegasil Prep ODS, Senshu Co., Tokyo, Japan), with stepwise in 50%, 70%, 90% and 100% methanol. Each eluted fraction was tested with crustacean lethal toxicity. The most toxic fraction eluted with 90% methanol (1220 mg dry wt.) was purified by reversed phase liquid chromatography on a 20 × 250 mm column (SHISEIDO CAPCELL PAK-ODS, Shiseido Co., Tokyo, Japan) using an isocratic HPLC system (flow rate; 4 mL/min, detection; 190–800 nm) with 82% MeOH to yield the main toxic peak which was consisted with at least two compounds. A recycle chromatography HPLC system (system unit; Senshu Scientific SSC-1310 (Senshu Co., Tokyo, Japan), column; SHISEIDO CAPCELL PAK-ODS (Shiseido Co., Tokyo, Japan) 10 × 250 mm, solvent; 82% MeOH, flow rate; 1 mL/min, detection; 210 nm) was then utilized to completely isolate 12-*epi*-lyngbyatoxin A (**1**) (2.8 mg) and lyngbyatoxin A (**2**) (10.2 mg) from the main toxic fraction.

### 3.4. 12-Epi-Lyngbyatoxin A (1) and Lyngbyatoxin A (2)

12-*Epi*-lyngbyatoxin A (**1**): Gummy solid; [α]^d^_18_ = +85.5° (*c* 0.12, MeOH); Circular dichroism (CD, in MeOH), [θ]_212.5nm_ −2760, [θ]_215nm_ 970, [θ]_225nm_ 33,900, [θ]_235nm_ 41,200, [θ]_249.5nm_ 1650, [θ]_252.5*nm*_ −1650, [θ]_258nm_ −4100, [θ]_280nm_ −690, [θ]_310nm_ 1470, [θ]_330nm_ 630 (Figure 3); UV λ_max_ (EtOH) nm (log ε) 231 (4.33), 301 (3.86); ^1^H NMR (800 MHz, CDCl_3_; 600 MHz, CD_3_OD) and ^13^C NMR (200 MHz, CDCl_3_) data; HRESIMS *m*/*z* [M + H]^+^ 438.3070 (calcd for C_27_H_39_N_3_O_2_, 437.3042).

Lyngbyatoxin A (**2**): Gummy solid; [α]^d^_18_ = −102.4° (*c* 0.13, MeOH); Circular dichroism (CD, in MeOH), [θ]_215nm_ −660, [θ]_221.5nm_ −6550, [θ]_229.5nm_ 2670, [θ]_234nm_ 12,200, [θ]_237.5nm_ 2510, [θ]_257nm_ −20,100, [θ]_298nm_ −790, [θ]_313nm_ 5520, [θ]_333nm_ 470 (Figure 3); UV λ_max_ (EtOH) nm (log ε) 230 (4.46), 301 (3.98); ^1^H NMR (800 MHz, CDCl_3_) and ^13^C NMR (200 MHz, CDCl_3_) data.

### 3.5. Cytotoxicity Assay

The cytotoxic activities of compounds **1** and **2** were tested toward L1210 mouse leukemia cells. The detailed methods were reported previously [39]. Differences with the reported methods [39] were (a) samples were dissolved with methanol; and (b) the methanol dissolved samples were applied onto each well and air-dried before the addition of L1210 cells.

### 3.6. Crustacean Lethality Test

The shrimp *Palaemon paucidens* (average weight, 0.5 g) was used for the crustacean lethality test. Tested samples were suspended with 1% Tween 20 (polyoxyethylene (20) sorbitan monolaurate). Two micro liter of each sample suspension was injected into the abdominal cavity of the shrimp (*n* = 3–5). The shrimp were monitored every 30 min for 4 h. Through the lethality tests, the lethal dose 100% values (LD_100_) were determined. One percent Tween 20 without sample was used as reference. LD_100_ was defined as weight of sample per unit weight of crustacean.

### 3.7. Binding Assay of PKC Ligands Using PKC-C1B Peptide

The binding of [^3^H]PDBu to the PKCδ-C1B peptide was evaluated by the procedure of Sharkey and Blumberg [40] with modifications as reported previously [41,42] using 50 mM tris-maleate buffer (pH 7.4 at 4 °C), 13.8 nM PKCδ-C1B peptide, 20 nM [^3^H]PDBu (18.7 Ci/mmol, Perkin-Elmer Life Sciences), 50 μg/mL 1,2-dioleoyl-*sn*-glycero-3-phospho-l-serine (Sigma, St. Louis, MO, USA), 3 mg/mL bovine γ-globulin (Sigma, St. Louis, MO, USA), and various concentrations of inhibitors. The concentration of the properly folded PKCδ-C1B peptide was estimated to be about 3 nM on the basis of *B*_max_ values of Scatchard analyses reported previously [41,42]. Binding affinity was evaluated based on the concentration required to cause 50% inhibition of the specific binding of [^3^H]PDBu, the IC_50_, which was calculated with Microsoft Excel. The inhibition constant, *K_i_*, was calculated by the method of Sharkey and Blumberg [40].

## 4. Conclusions

The lethal toxicity guided purification of an ethyl acetate extract from the cyanobacterium *Moorea producens* resulted in the isolation of lyngbyatoxin A (**2**) and a new compound 12-*epi*-lyngbyatoxin A (**1**). The absolute configuration of compound **1** was deduced from the analyses of the NMR, optical rotation and CD spectra. While 12-*epi*-lyngbyatoxin A (**1**) and lyngbyatoxin A (**2**) showed comparable cytotoxic and crustacean lethal activities, 12-*epi*-lyngbyatoxin A (**1**) had more than 100 times lower binding affinity for protein kinase Cδ compared to lyngbyatoxin A (**2**). This suggests that the cytotoxic and lethal mechanism of lyngbyatoxin-type compounds might be mediated through a non-PKC activation pathway.

## Supplementary Files

Supplementary File 1 Supplementary Information (PDF, 1823 KB)

## References

[1] Engene N., Rottacker E.C., Kaštovský J., Byrum T., Choi H., Ellisman M.H., Komárek J., Gerwick W.H. (2012). *Moorea producens* gen. nov., sp. nov. and *Moorea bouillonii* comb. nov., tropical marine cyanobacteria rich in bioactive secondary metabolites. Int. J. Syst. Evol. Microbiol..

[2] Burja A.M., Banaigs B., Abou-Mansour E., Grant Burgess J., Wright P.C. (2001). Marine cyanobacteria—Aprolific source of natural products. Tetrahedron.

[3] Cardellina J.H., Marner F.J., Moore R.E. (1979). Seaweed dermatitis: Structure of lyngbyatoxin A. Science.

[4] Moore R.E., Blackman A.J., Cheuk C.E., Mynderse J.S., Matsumoto G.K., Clardy J., Woodard R.W., Craig J.C. (1984). Absolute stereochemistries of the aplysiatoxins and oscillatoxin A. J. Org. Chem..

[5] Mynderse J.S., Moore R.E., Kashiwagi M., Norton T.R. (1977). Antileukemia activity in the Osillatoriaceae: Isolation of debromoaplysiatoxin from *Lyngbya*. Science.

[6] Fujiki H., Mori M., Nakayasu M., Terada M., Sugimura T., Moore R.E. (1981). Indole alkaloids: Dihydroteleocidin B, teleocidin, and lyngbyatoxin A as members of a new class of tumor promoters. Proc. Natl. Acad. Sci. USA.

[7] Fujiki H., Tanaka Y., Miyake R., Kikkawa U., Nishizuka Y., Sugimura T. (1984). Activation of calcium-activated, phospholipid-dependent protein kinase (protein kinase C) by new classes of tumor promoters: Teleocidin and debromoaplysiatoxin. Biochem. Biophys. Res. Commun..

[8] Yasumoto T. (1998). Fish poisoning due to toxins of microalgal origins in the Pacific. Toxicon.

[9] Ito E., Satake M., Yasumoto T. (2002). Pathological effects of lyngbyatoxin A upon mice. Toxicon.

[10] Nagai H., Yasumoto T., Hokama Y. (1996). Aplysiatoxin and debromoaplysiatoxin as the causative agents of a red alga *Gracilaria. coronopifolia* poisoning in Hawaii. Toxicon.

[11] Nagai H., Yasumoto T., Hokama Y. (1997). Manauealides, some of the causative agents of a red alga *Gracilaria. coronopifolia* poisoning in Hawaii. J. Nat. Prod..

[12] Nagai H., Kan Y., Fujita T., Sakamoto B., Hokama Y. (1998). Manauealide C and anhydrodebromoaplysiatoxin, toxic constituents of the Hawaiian red alga, *Gracilaria Coronopifolia*. Biosci. Biotechnol. Biochem..

[13] Arthur K., Limpus C., Balazs G., Capper A., Udy J., Shaw G., Keuper-Bennett U., Bennett P. (2008). The exposure of green turtles (*Chelonia mydas*) to tumour promoting compounds produced by the cyanobacterium *Lyngbya. majuscula* and their potential role in the aetiology of fibropapillomatosis. Harmful Algae.

[14] Harr K.E., Szabo N.J., Cichra M., Phlips J.E. (2008). Debromoaplysiatoxin in *Lyngbya*-dominated mats on manatees (*Trichechus manatus latirostris*) in the Florida King’s Bay ecosystem. Toxicon.

[15] Namikoshi M., Rinehart K.L. (1996). Bioactive compounds produced by cyanobacteria. J. Ind. Microbiol..

[16] Gademann K., Portmann C. (2008). Secondary metabolites from cyanobacteria: Complex structures and powerful bioactivities. Curr. Org. Chem..

[17] Molinski T.F., Dalisay D.S., Lievens S.L., Saludes J.P. (2008). Drug development from marine natural products. Nat. Rev. Drug. Discov..

[18] Sakai S.I., Hitotsuyanagi Y., Aimi N., Fujiki H., Suganuma M., Sugimura T., Endo Y., Shudo K. (1986). Absolute configuration of lyngbyatoxin A (teleocidin A-1) and teleocidin A-2. Tetrahedron Lett..

[19] Aimi N., Odaka H., Sakai S.I., Fujiki H., Suganuma M., Moore R.E., Patterson G.M.L. (1990). Lyngbyatoxins B and C, two new irritants from *Lyngbya majuscula*. J. Nat. Prod..

[20] Muratake H., Okabe K., Natsume M. (1991). Synthesis of teleocidins A, B and their congeners. Part 2. Synthesis of lyngbyatoxin A (teleocidin A-1), teleocidin A-2, pendolmycin, and (*R*,*E*)- and (*S*,*E*)-7-(3,7,11-trimethyl-1,6,10-dodecatrien-3-yl)-(−)-indolactams V. Tetrahedron.

[21] Endo Y., Shudo K., Okamoto T. (1982). Molecular requirements for epigenetic modulators. Synthesis of active fragments of teleocidins and lyngbyatoxin. Chem. Pharm. Bull..

[22] Irie K., Hirota M., Hagiwara N., Koshimizu K., Hayashi H., Murao S., Tokuda H., Ito Y. (1984). The Epstein-Barr virus early antigen inducing indole alkaloids, (−)-indolactam-V and its related compounds, produced by *Actinomycetes*. Agric. Biol. Chem..

[23] Fujiki H., Suganuma M., Nakayasu M., Tahira T., Endo Y., Shudo K., Sugimura T. (1984). Structure-activity studies on synthetic analogues (indolactams) of the tumor promoter teleocidin. Jpn. J. Cancer Res..

[24] Kawai T., Ichinose T., Takeda M., Tomioka N., Endo Y., Yamaguchi K., Shudo K., Itai A. (1992). Prediction of ring conformations of indolactams. Crystal and solution structures. J. Org. Chem..

[25] Sakai S.I., Aimi N., Yamaguchi K., Hitotsuyanagi Y., Watanabe C., Yokose K., Koyama Y., Shudo K., Itai A. (1984). Elucidation of the structure of olivoretin A and D (teleocidin B). Chem. Pharm. Bull..

[26] Endo Y., Shudo K., Furuhata K., Ogura H., Sakai S.I., Aimi N., Hitotsuyanagai Y., Koyama Y. (1984). Synthesis of optically active teleocidin derivatives. Absolute configuration of teleocidin B and olivoretin A. Chem. Pharm. Bull..

[27] Hitotsuyanagi Y., Yamaguchi K., Ogata K., Aimi N., Sakai S.I., Koyama Y., Endo Y., Shudo K., Itai A., Iitaka Y. (1984). Elucidation of the structures of olivoretin B and C. Chem. Pharm. Bull..

[28] Endo Y., Shudo K., Itai A., Hasegawa M., Sakai S.I. (1986). Synthesis and stereochemistry of indolactam-V, an active fragment of teleocidins. Structural requirements for tumor-promoting activity. Tetrahedron.

[29] Fujiki H., Sugimura T. (1987). New classes of tumor promoters: Teleocidin, aplysiatoxin, and palytoxin. Adv. Cancer Res..

[30] Irie K., Kajiyama S.-I., Funaki A., Koshimizu K., Hayashi H., Arai M. (1990). Biosynthesis of indole alkaloid tumor promoter teleocidins (I): Possible biosynthetic pathway of the monoterpenoid moieties of teleocidins. Tetrahedron.

[31] Irie K., Isaka T., Iwata Y., Yanai Y., Nakamura Y., Koizumi F., Ohigashi H., Wender P.A., Satomi Y., Nishino H. (1996). Synthesis and biological activities of new conformationally restricted analogues of (−)-indolactam-V: Elucidation of the biologically active conformation of the tumor-promoting teleocidins. J. Am. Chem. Soc..

[32] Izumikawa M., Khan S.T., Komaki H., Takagi M., Shin-ya K. (2009). JBIR-31, a new teleocidin analog, produced by salt-requiring *Streptomyces* sp. NBRC 105896 isolated from a marine sponge. J. Antibiot..

[33] Pu J., Deng K., Butera J., Chlenov M., Gilbert A., Kagan M., Mattes J., Resnick L. (2010). *De novo* synthesis of teleocidin B analogs. Tetrahedron.

[34] Irie K., Hagiwara N., Koshimizu K. (1987). New probes for receptor analysis of tumor promoters synthesis of fluorescent derivatives of (−)-indolactam V, the basic ring-structure of teleocidins. Tetrahedron.

[35] Nakagawa Y., Yanagita R.C., Hamada N., Murakami A., Takahashi H., Saito N., Nagai H., Irie K. (2009). A simple analogue of tumor-promoting aplysiatoxin is an antineoplastic agent rather than a tumor promoter: Development of a synthetically accessible protein kinase C activator with bryostatin-like activity. J. Am. Chem. Soc..

[36] Yanagita R.C., Kamachi H., Tanaka K., Murakami A., Nakagawa Y., Tokuda H., Nagai H., Irie K. (2010). Role of the phenolic hydroxyl group in the biological activities of simplified analogue of aplysiatoxin with antiproliferative activity. Bioorg. Med. Chem. Lett..

[37] Kikumori M., Yanagita R.C., Tokuda H., Suzuki N., Nagai H., Suenaga K., Irie K. (2012). Structure-activity studies on the spiroketalmoiety of a simplified analogue of debromoaplysiatoxin with antiproliferative activity. J. Med. Chem..

[38] Hanaki Y., Kikumori M., Ueno S., Tokuda H., Suzuki N., Irie K. (2013). Structure-activity studies at position 27 of aplog-1, a simplified analog of debromoaplysiatoxin with anti-proliferative activity. Tetrahedron.

[39] Kawabata T., Lindsay D.J., Kitamura M., Konishi S., Nishikawa J., Nishida S., Kamio M., Nagai H. (2013). Evaluation of the bioactivities of water-soluble extracts from twelve deep-sea jellyfish species. Fish. Sci..

[40] Sharkey N.A., Blumberg P.M. (1985). Highly lipophilic phorbolesters as inhibitors of specific [^3^H]phorbol 12,13-dibutyrate binding. Cancer Res..

[41] Irie K., Oie K., Nakahara A., Yanai Y., Ohigashi H., Wender P.A., Fukuda H., Konishi H., Kikkawa U. (1998). Molecular basis for protein kinase C isozyme-selective binding: The synthesis, folding, and phorbol ester binding of the cysteine-rich domains of all protein kinase C isozymes. J. Am. Chem. Soc..

[42] Shindo M., Irie K., Nakahara A., Ohigashi H., Konishi H., Kikkawa U., Fukuda H., Wender P.A. (2001). Toward the identification of selective modulators of protein kinase C (PKC) isozymes: Establishment of a binding assay for PKC isozymes using synthetic C1 peptide receptors and identification of the critical residues involved in the phorbolester binding. Bioorg. Med. Chem..

