# Supplementary material for: A New Lyngbyatoxin from the Hawaiian Cyanobacterium Moorea producens
Source: Mar Drugs. 2014 May 12;12(5):2748–59. doi: 10.3390/md12052748 (PMC4052313; doi:10.3390/md12052748)
Supplement: Supplementary File 1 — Supplementary Information (PDF, 1823 KB) [file marinedrugs-12-02748-s001.pdf]

Supplementary Information

Figure S1. HR-ESI-MS spectrum of compound **1**.

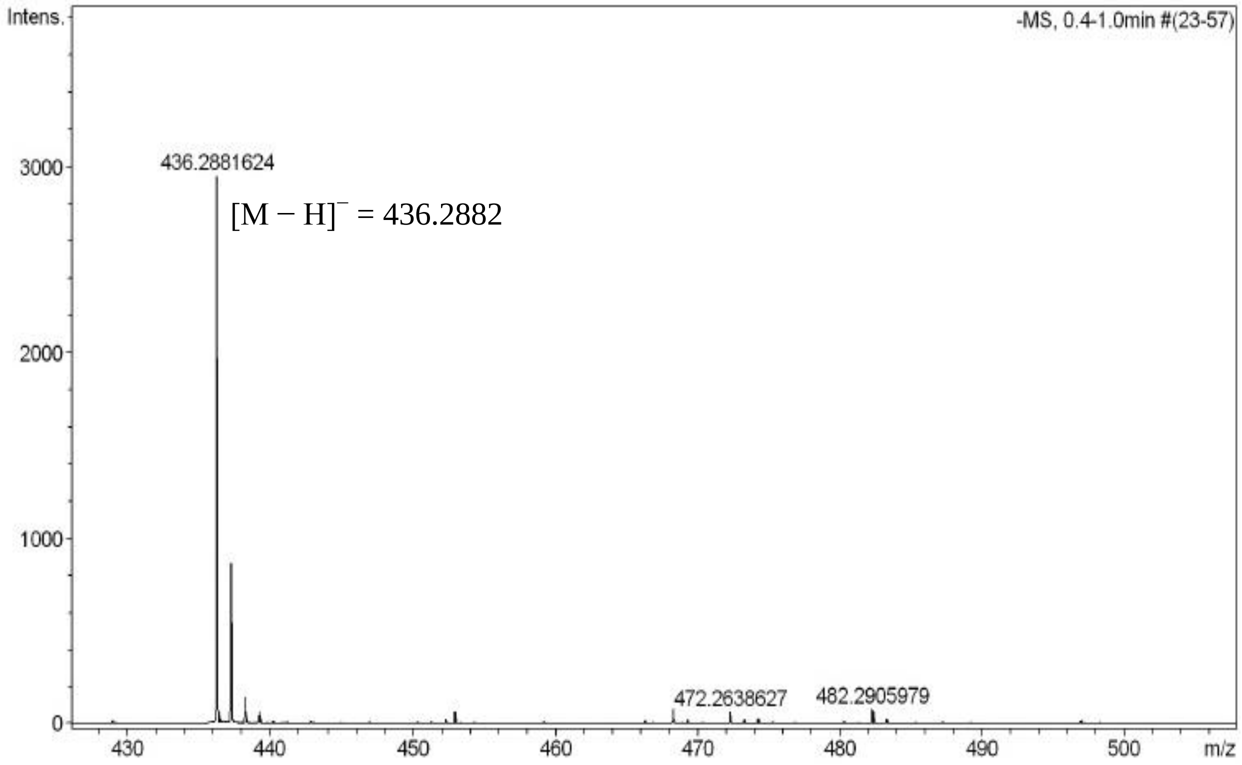

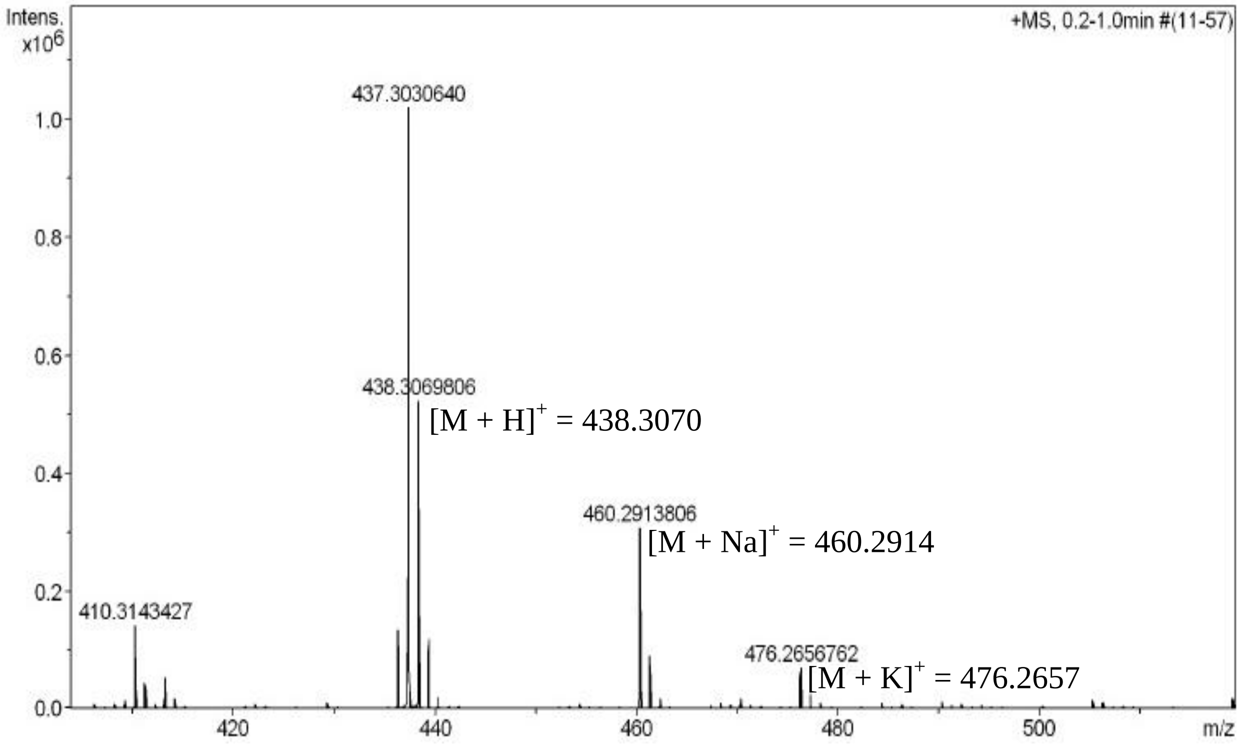

Figure S2. ^{1}H-NMR spectrum (800 MHz) of compound **1**, in $CDCl_3$.

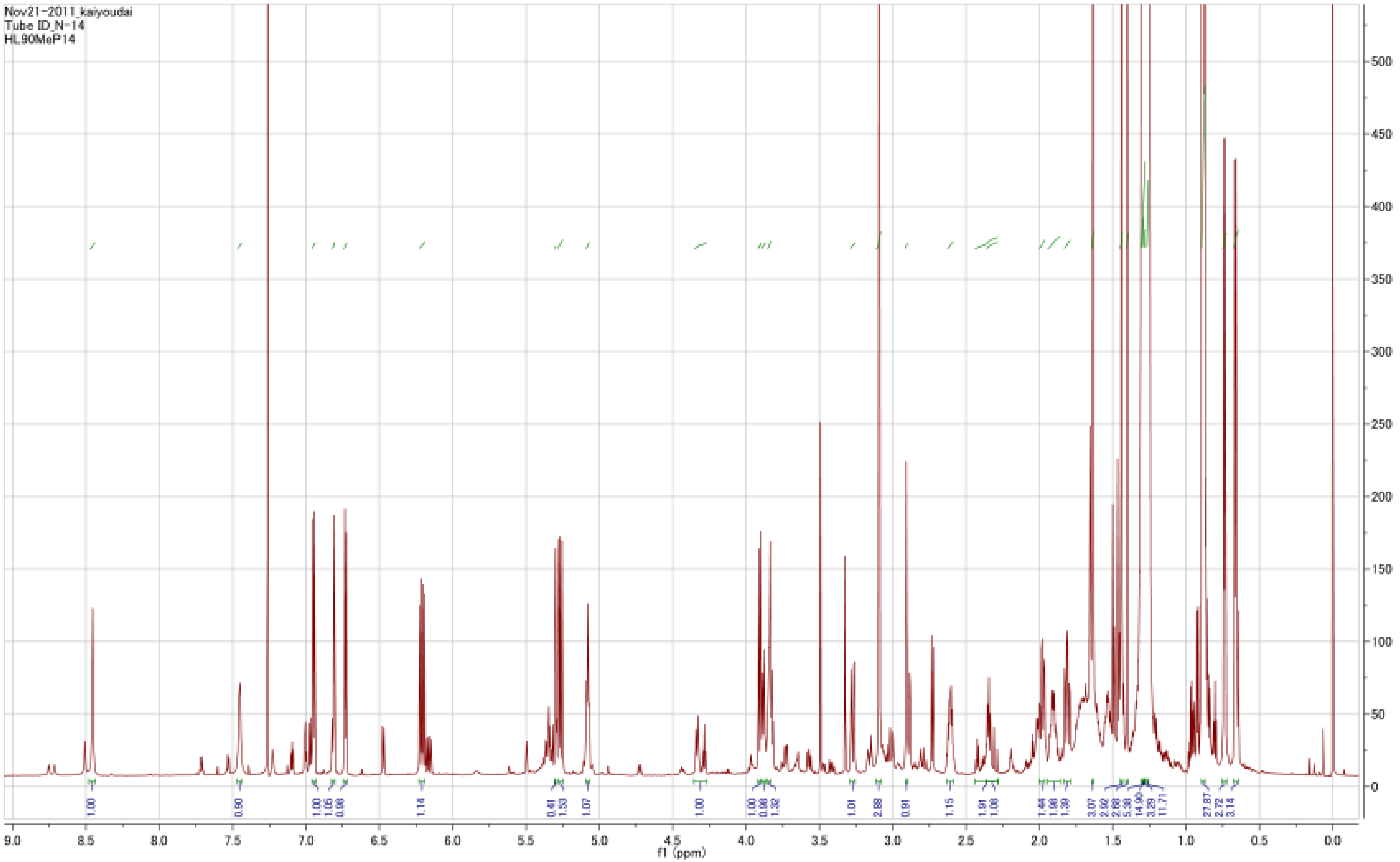

Figure S3. ^{13}C-NMR spectrum (200 MHz) of compound **1**, in $CDCl_3$.

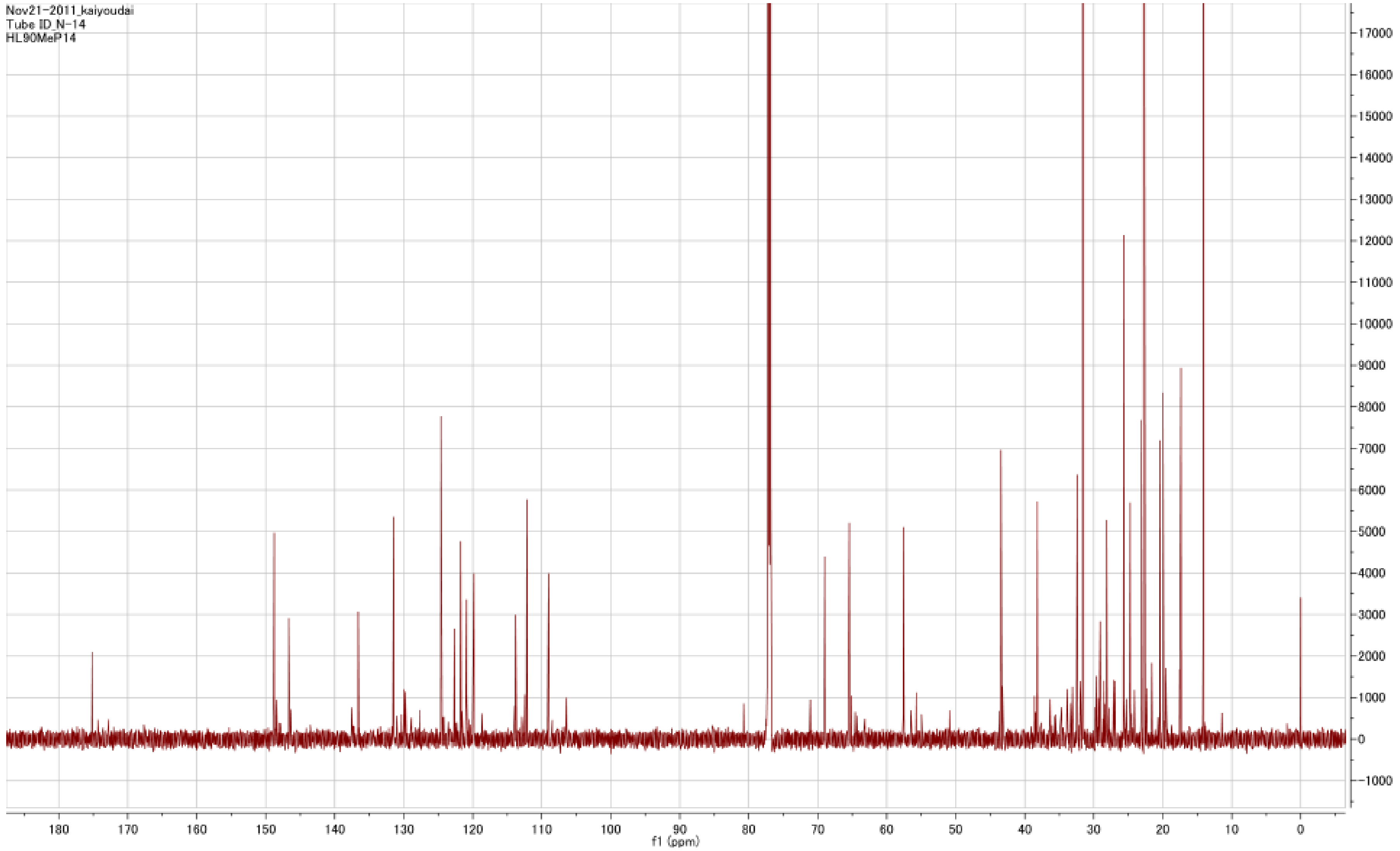

Figure S4. ^{1}H-^{1}H COSY spectrum of compound **1**, in $CDCl_3$.

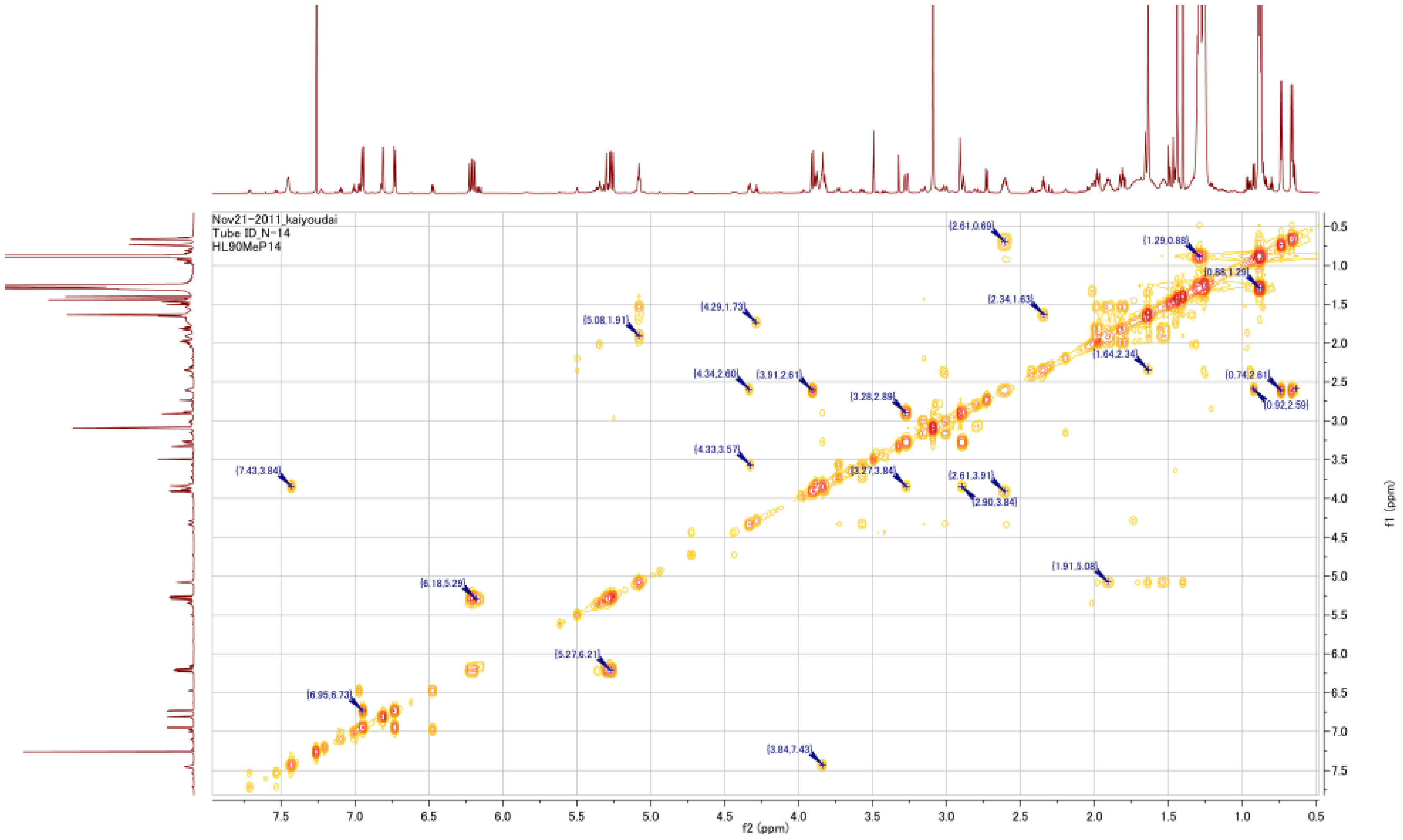

Figure S5. ^{1}H-^{13}C HSQC spectrum of compound **1**, in $CDCl_3$.

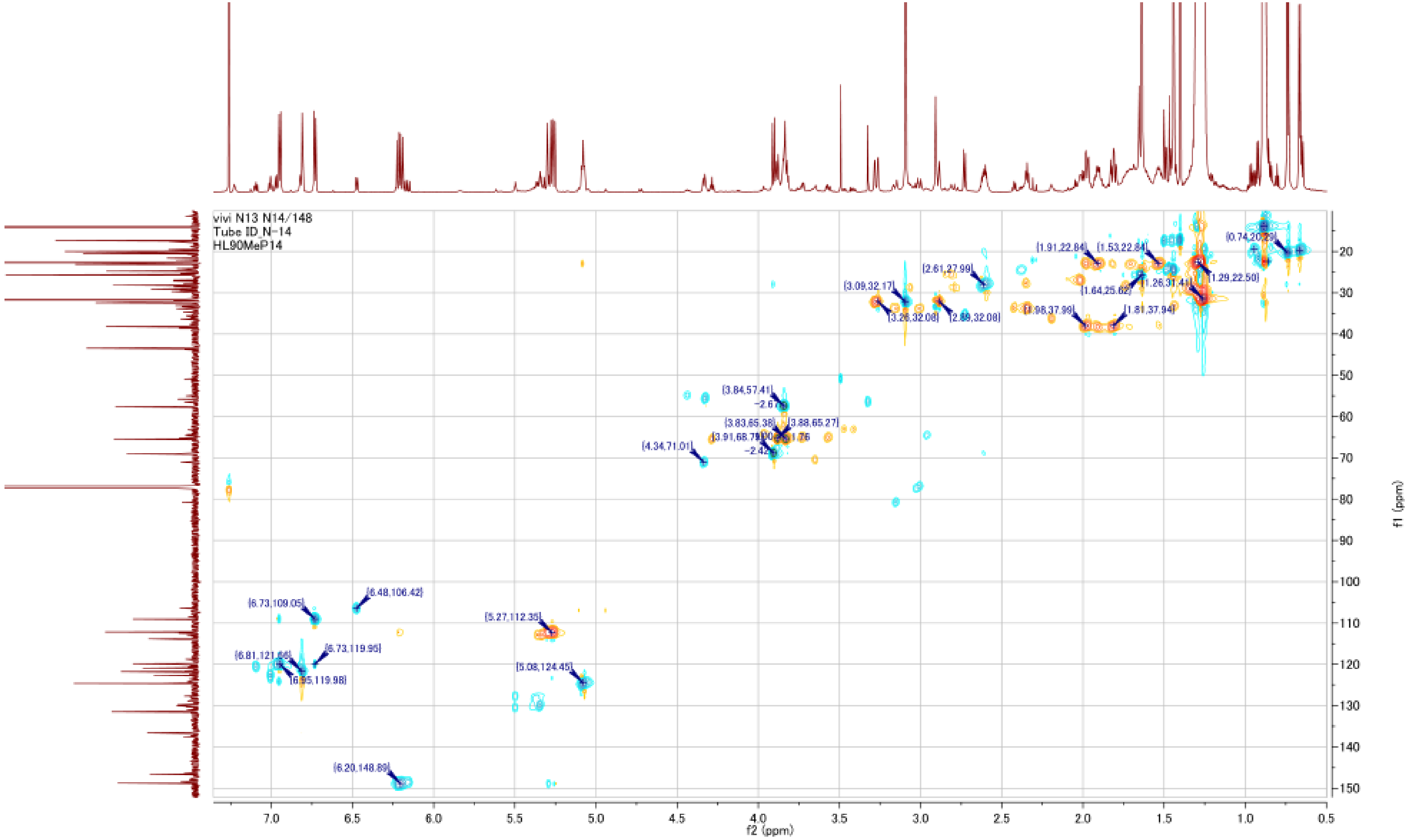

Figure S6. ^{1}H-^{13}C HMBC spectrum of compound **1**, in $CDCl_3$.

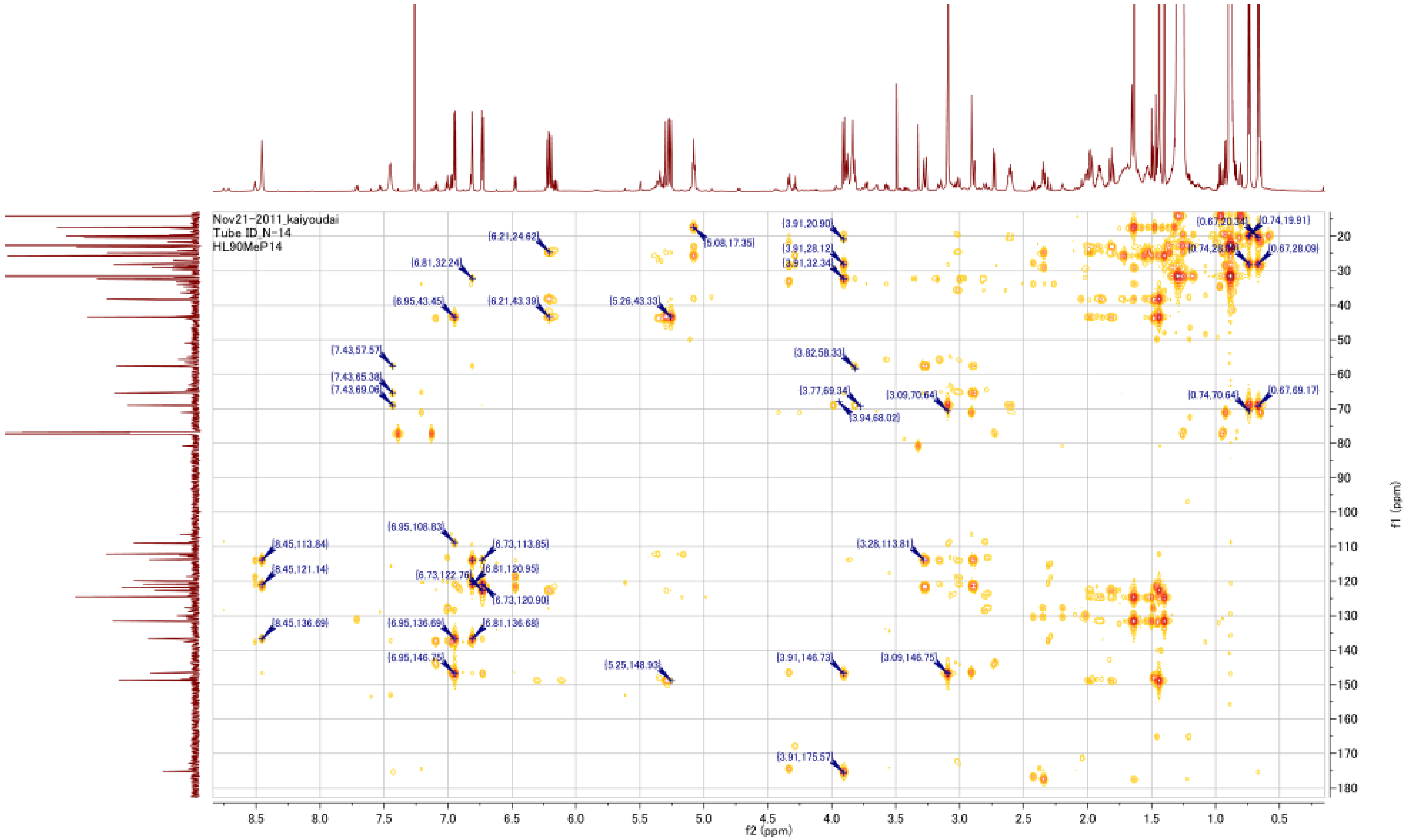

Figure S7. ^1H-^1H NOESY spectrum of compound **1**, in $CDCl_3$.

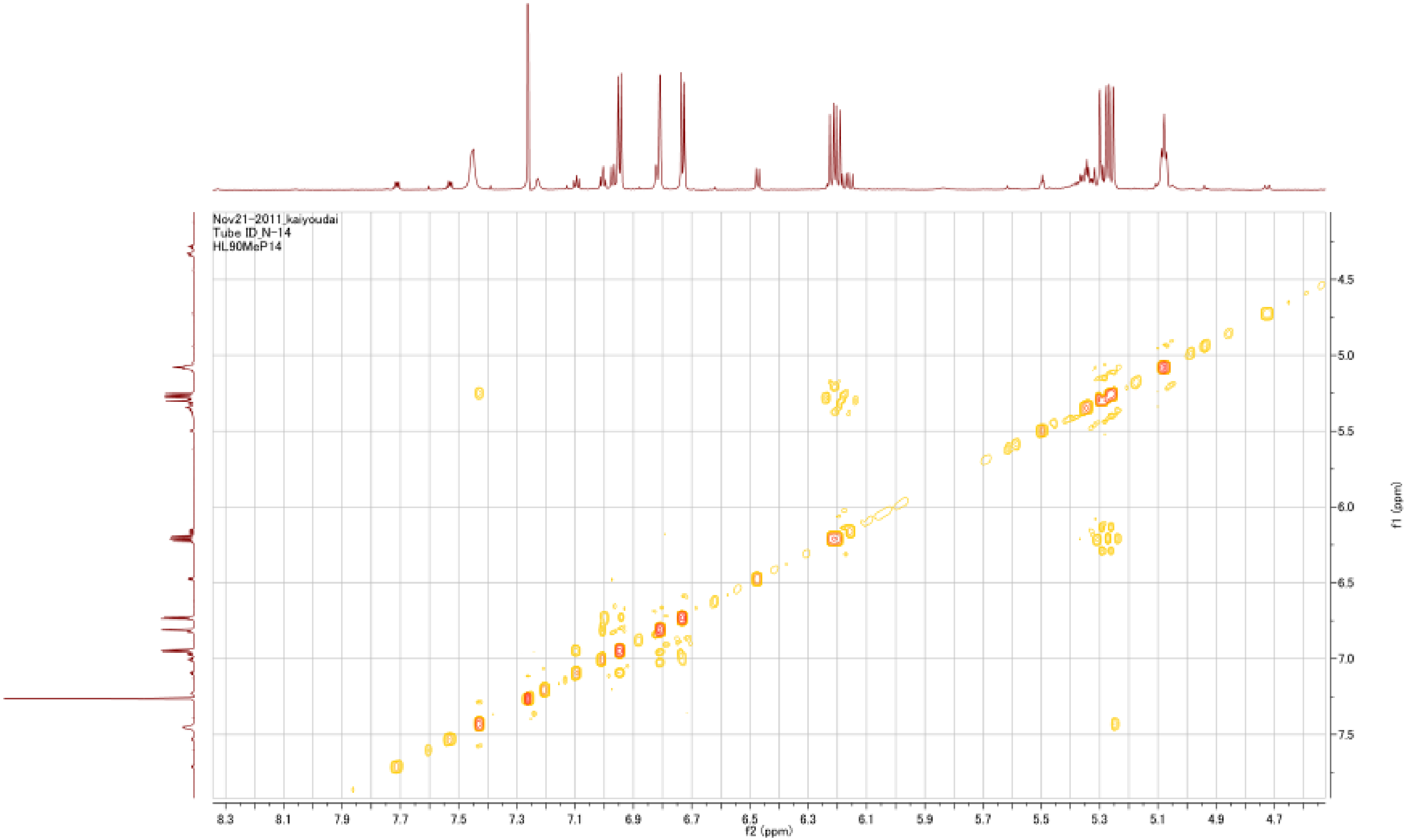

Figure S8. DEPT 45 spectrum of compound **1**, in $CDCl_3$.

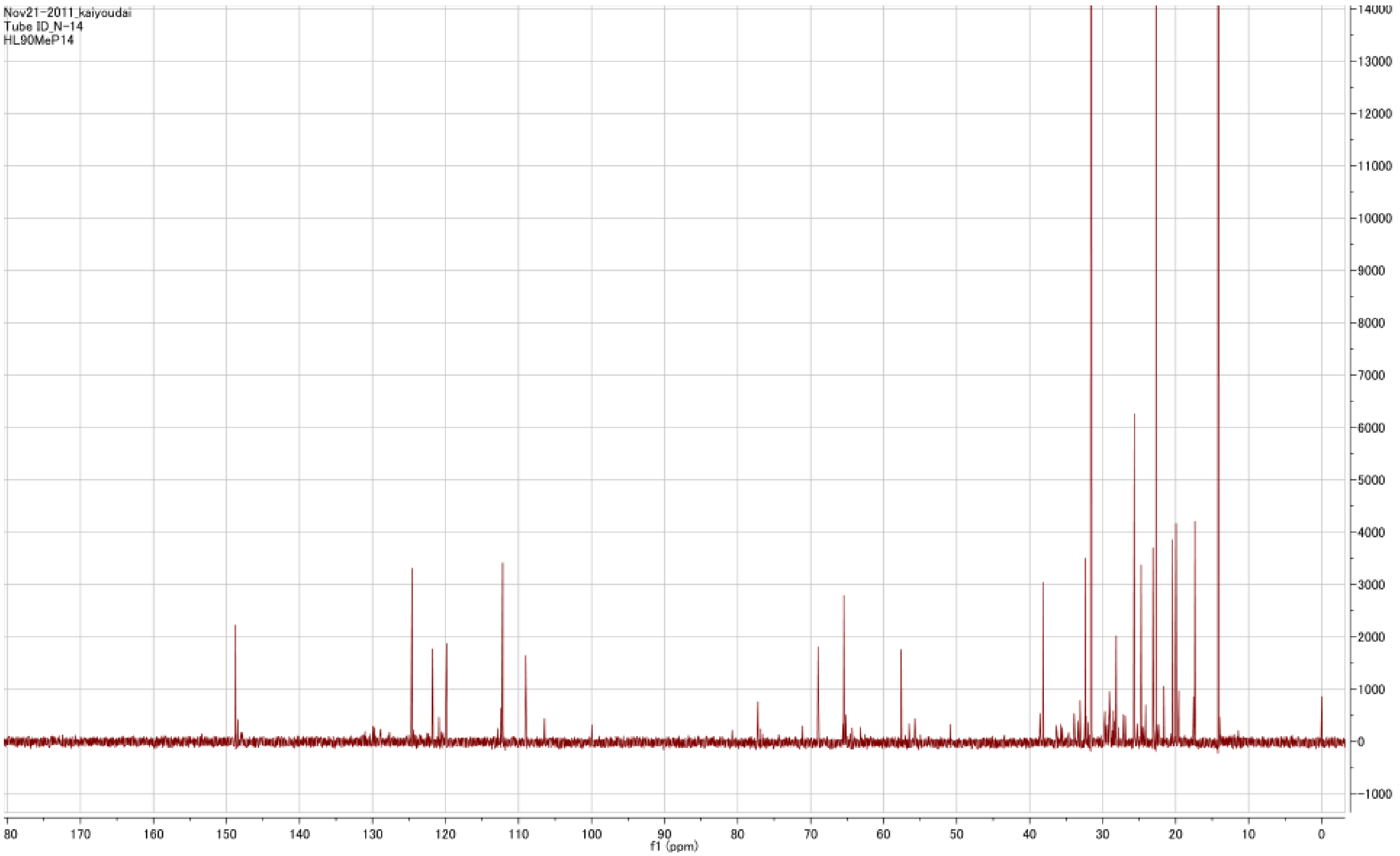

Figure S9. DEPT 90 spectrum of compound **1**, in $CDCl_3$.

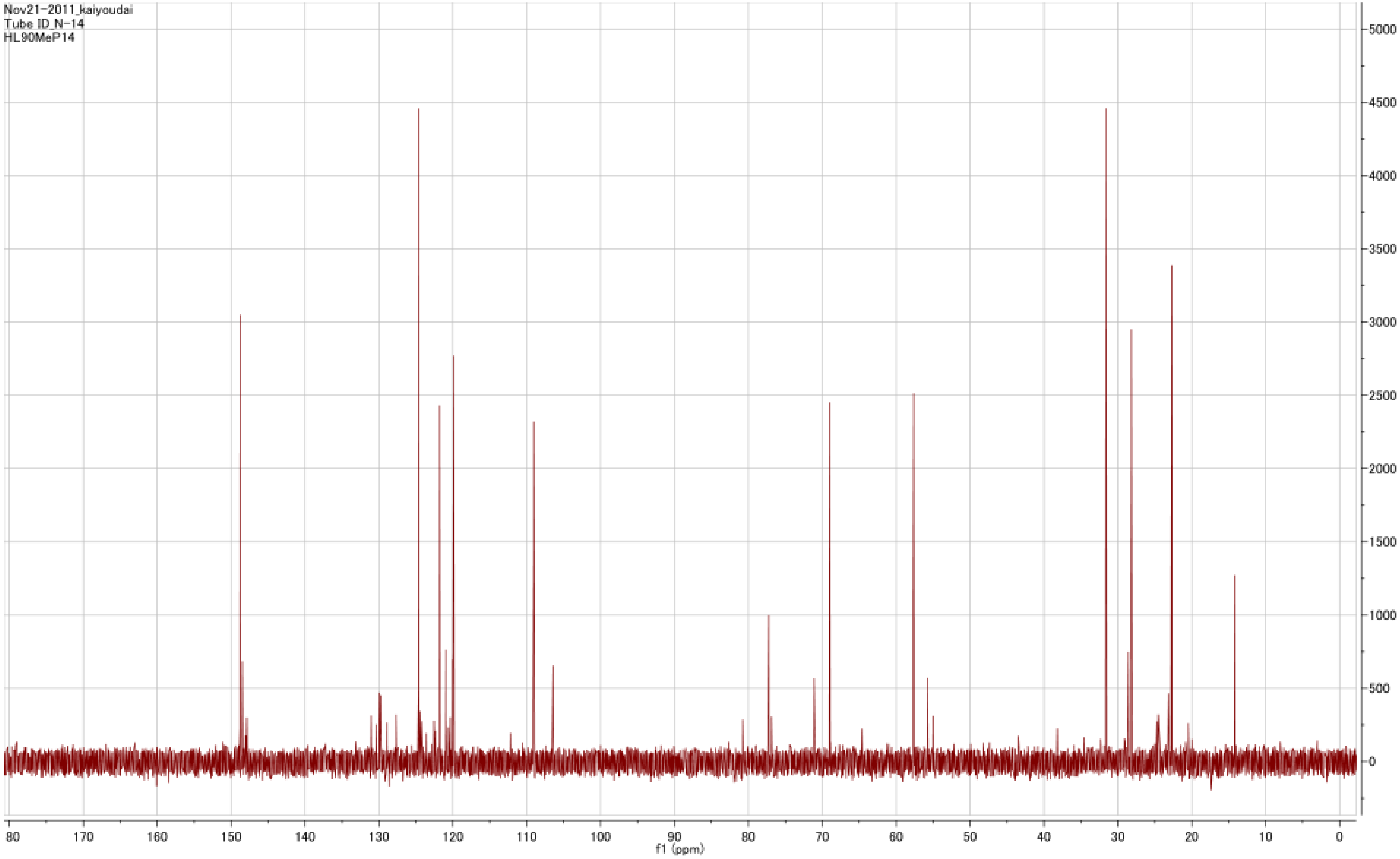

Figure S10. DEPT 135 spectrum of compound **1**, in $CDCl_3$.

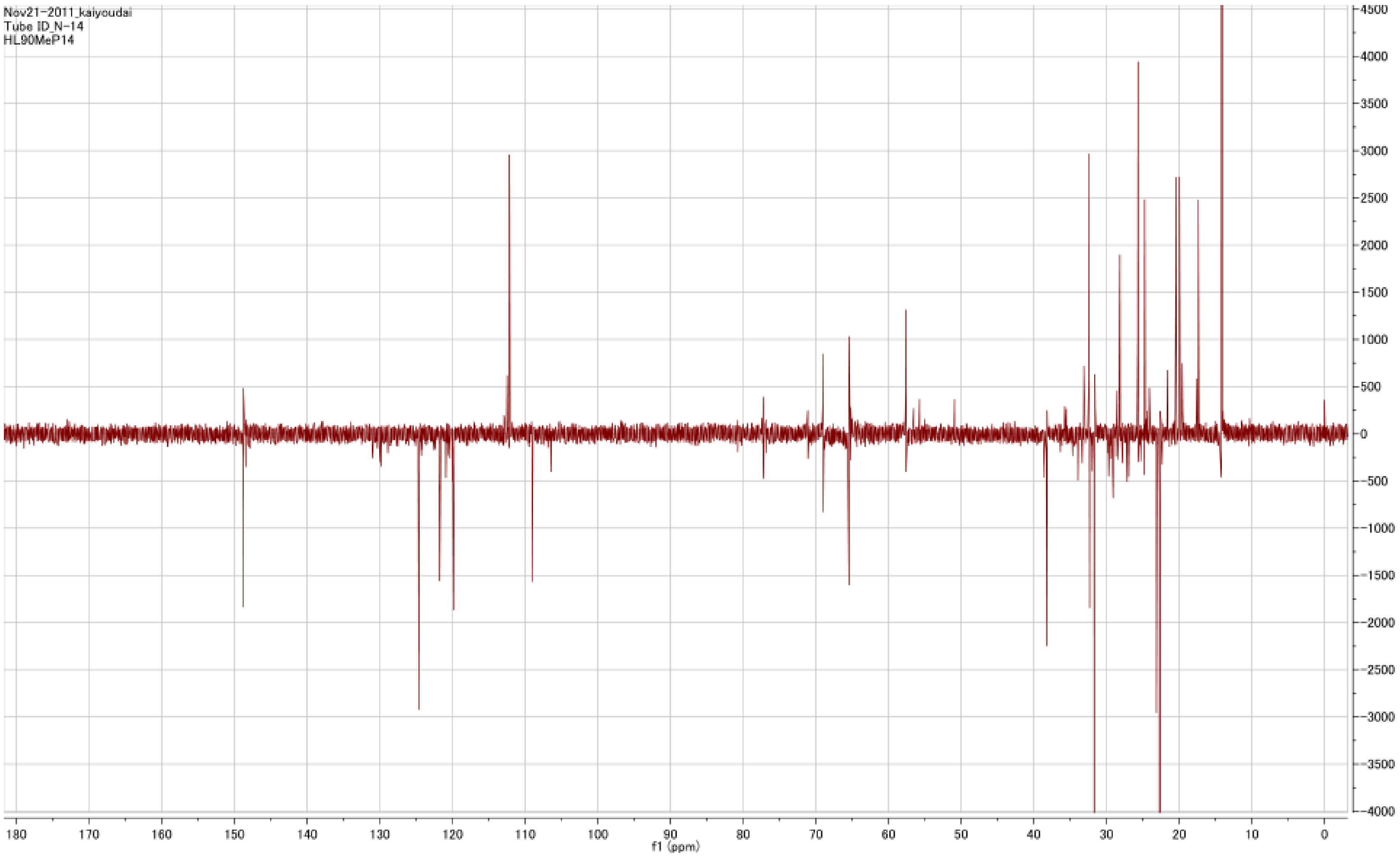

Figure S11. UV spectrum of compound **1**.

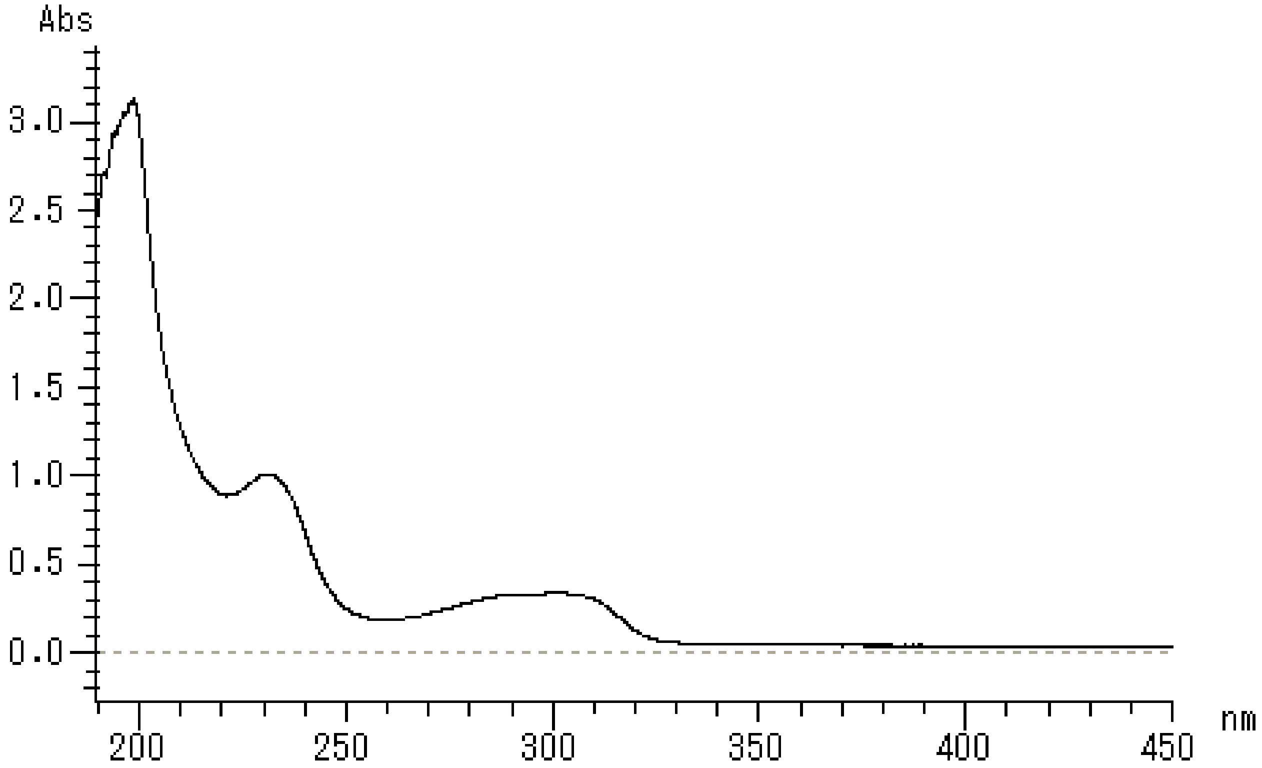

Figure S12. ^{1}H-NMR spectrum (800 MHz) of compound **2**, in $CDCl_3$.

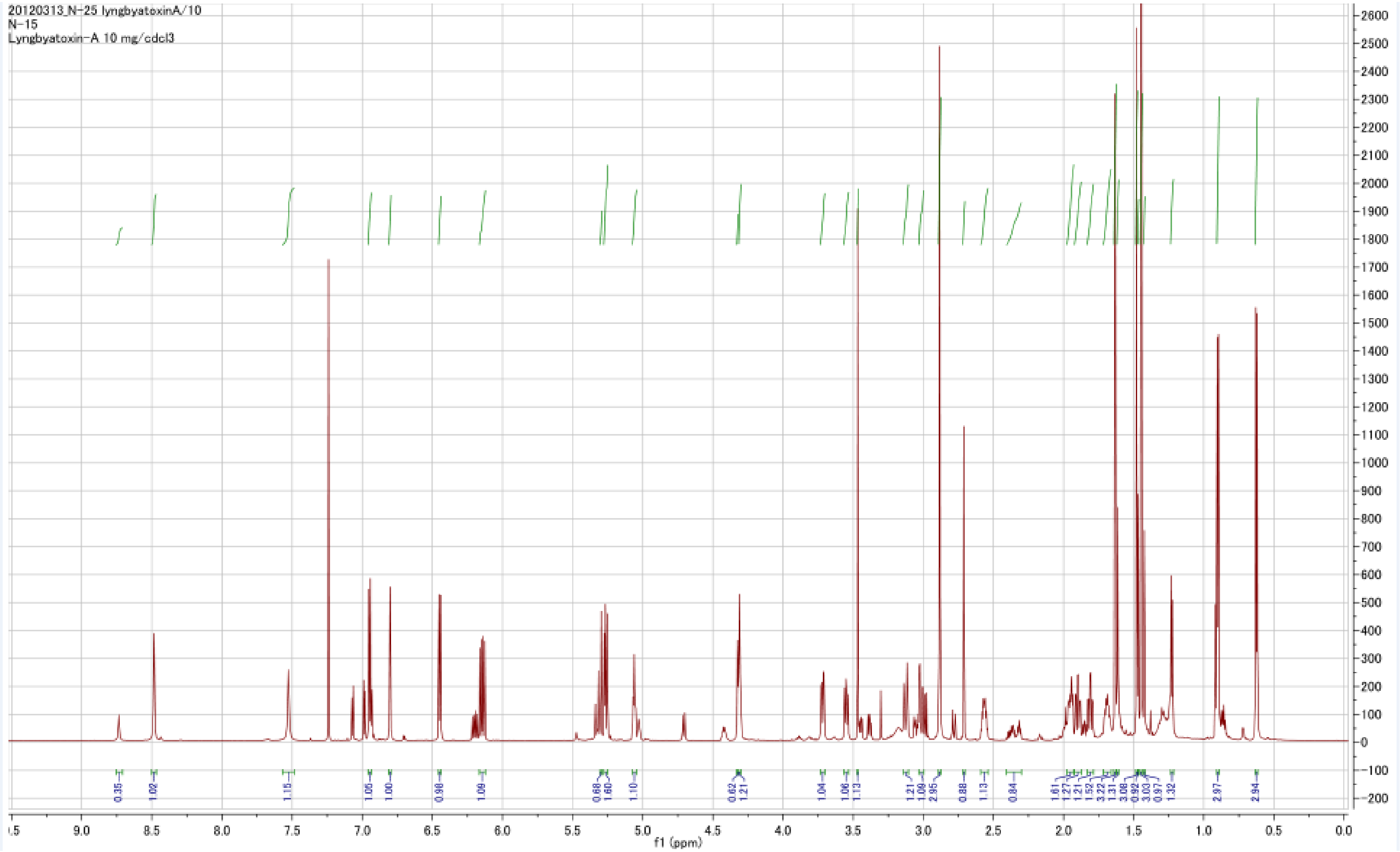

Figure S13. ^{13}C-NMR spectrum (200 MHz) of compound **2**, in $CDCl_3$.

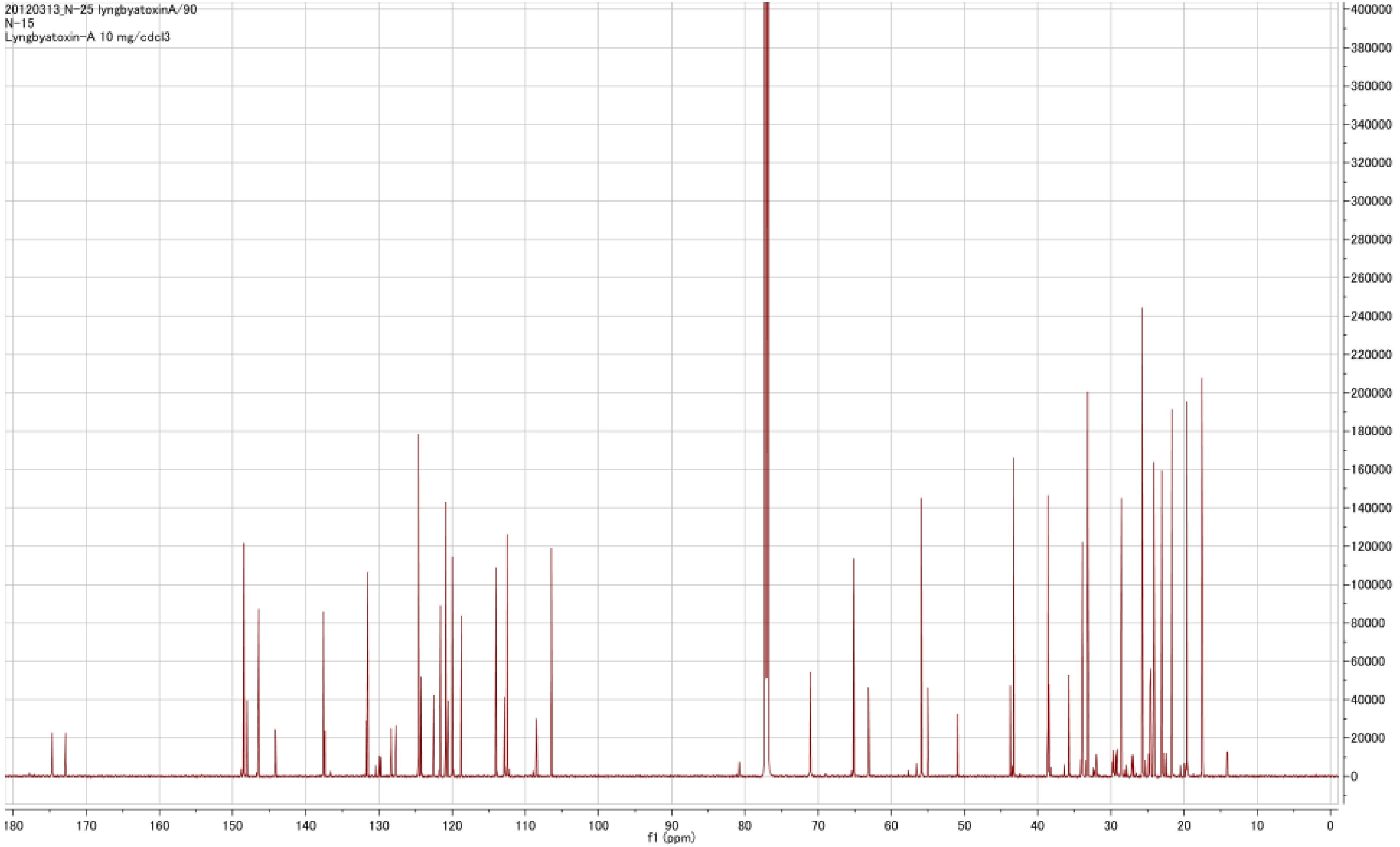

Figure S14. ^{1}H-^{1}H COSY spectrum of compound **2**, in $CDCl_3$.

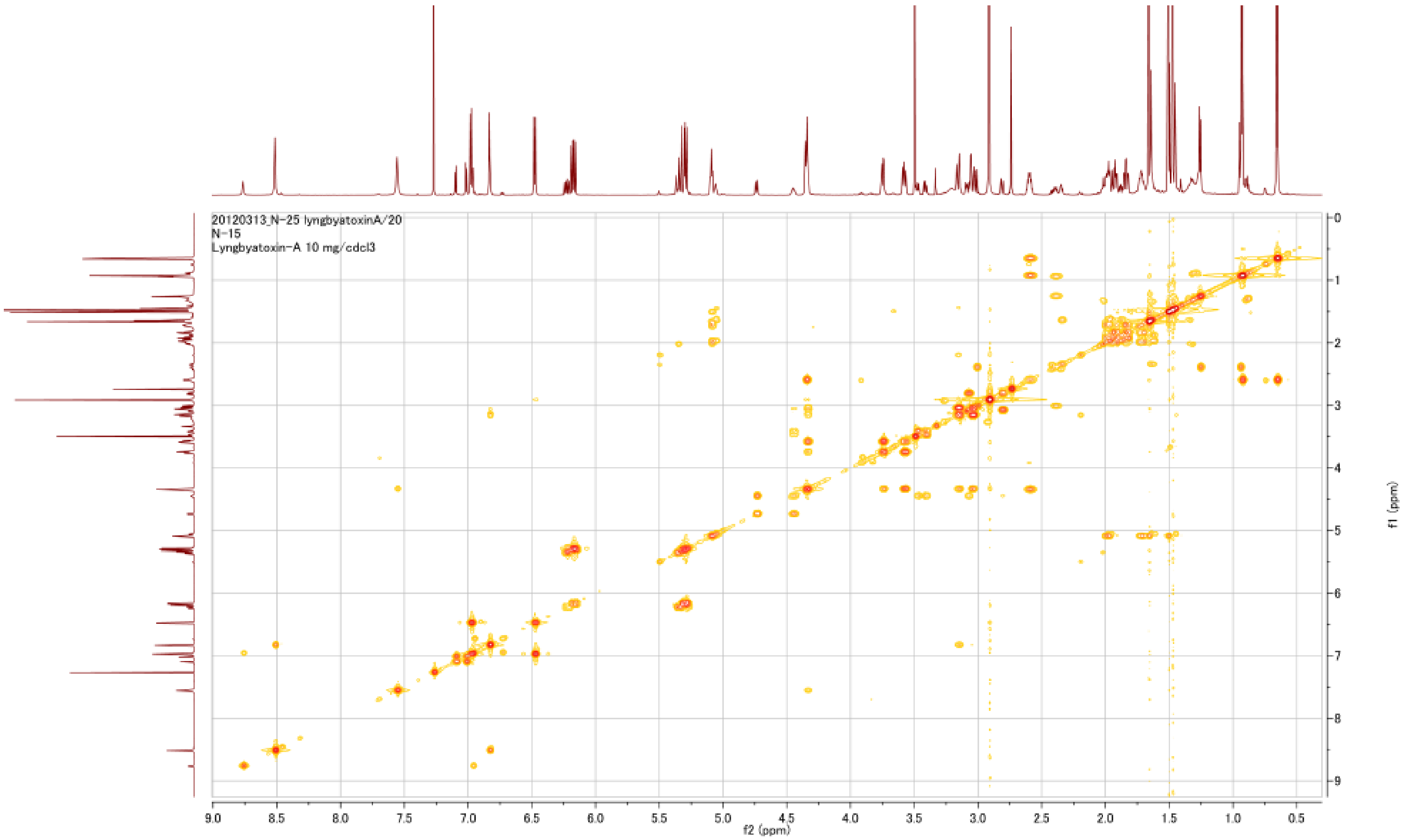

Figure S15. ^{1}H-^{13}C HSQC spectrum of compound **2**, in $CDCl_3$.

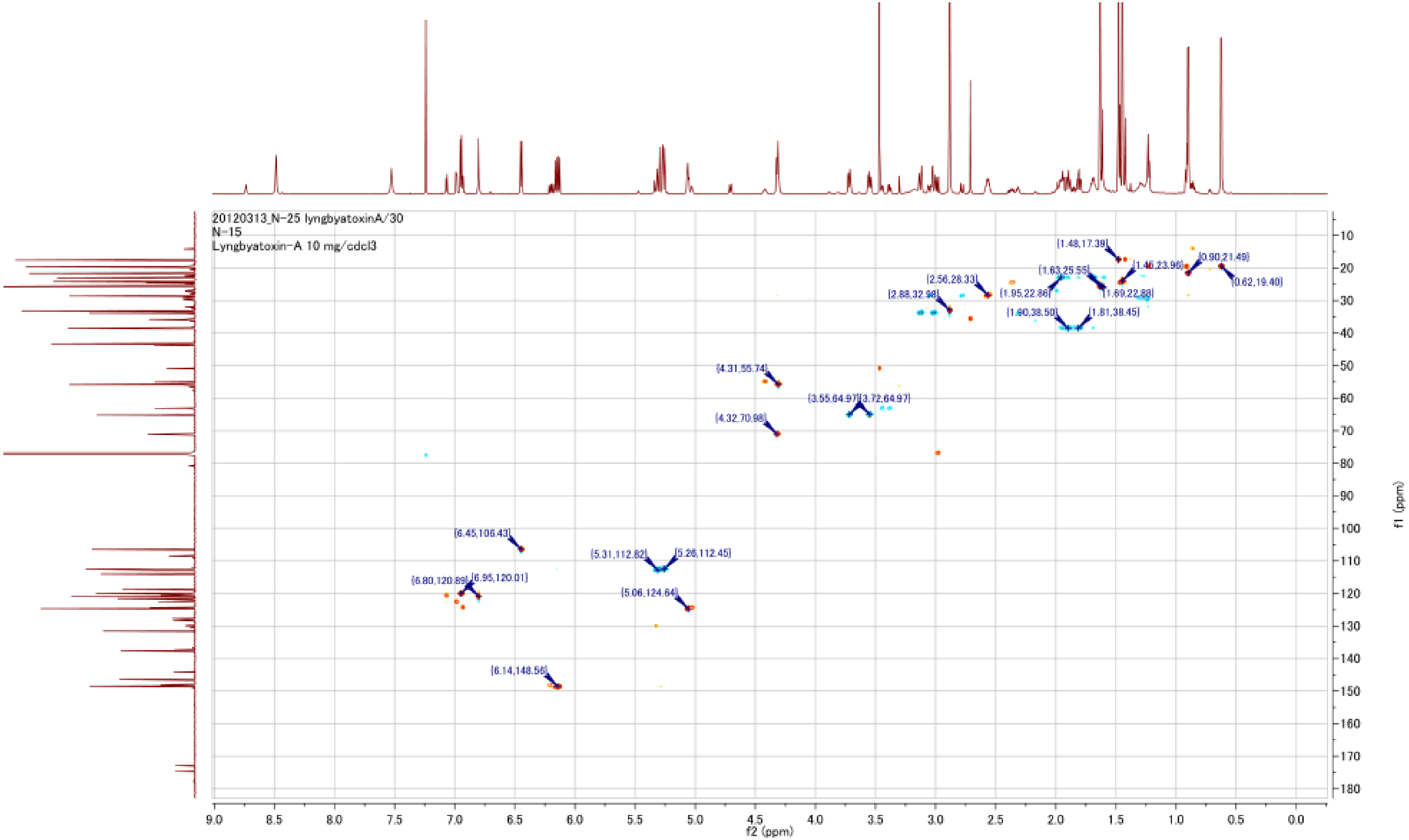

Figure S16. ^1H-^{13}C HMBC spectrum of compound **2**, in $CDCl_3$.

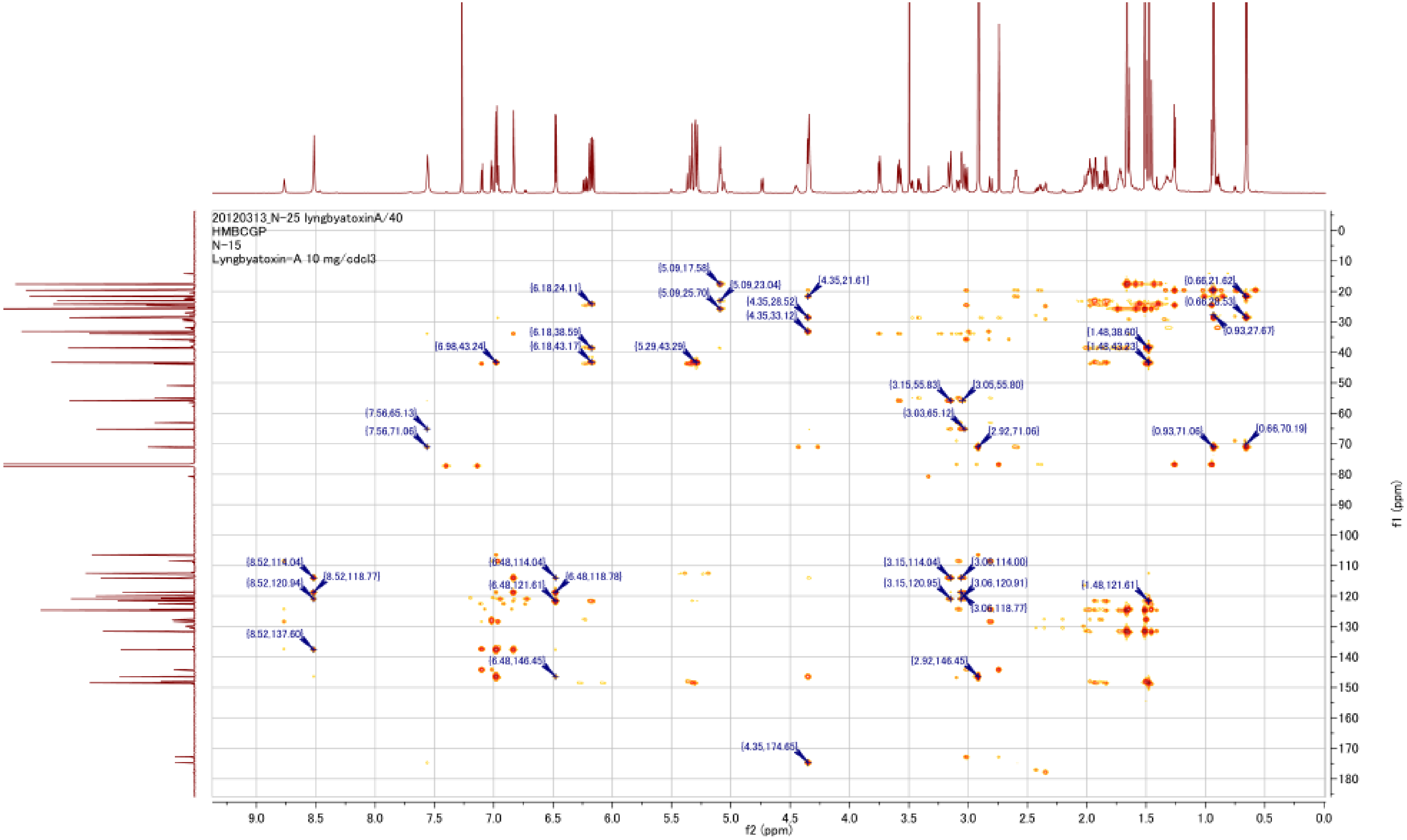

Figure S17. ^{1}H-^{1}H NOESY spectrum of compound **2**, in $CDCl_3$.

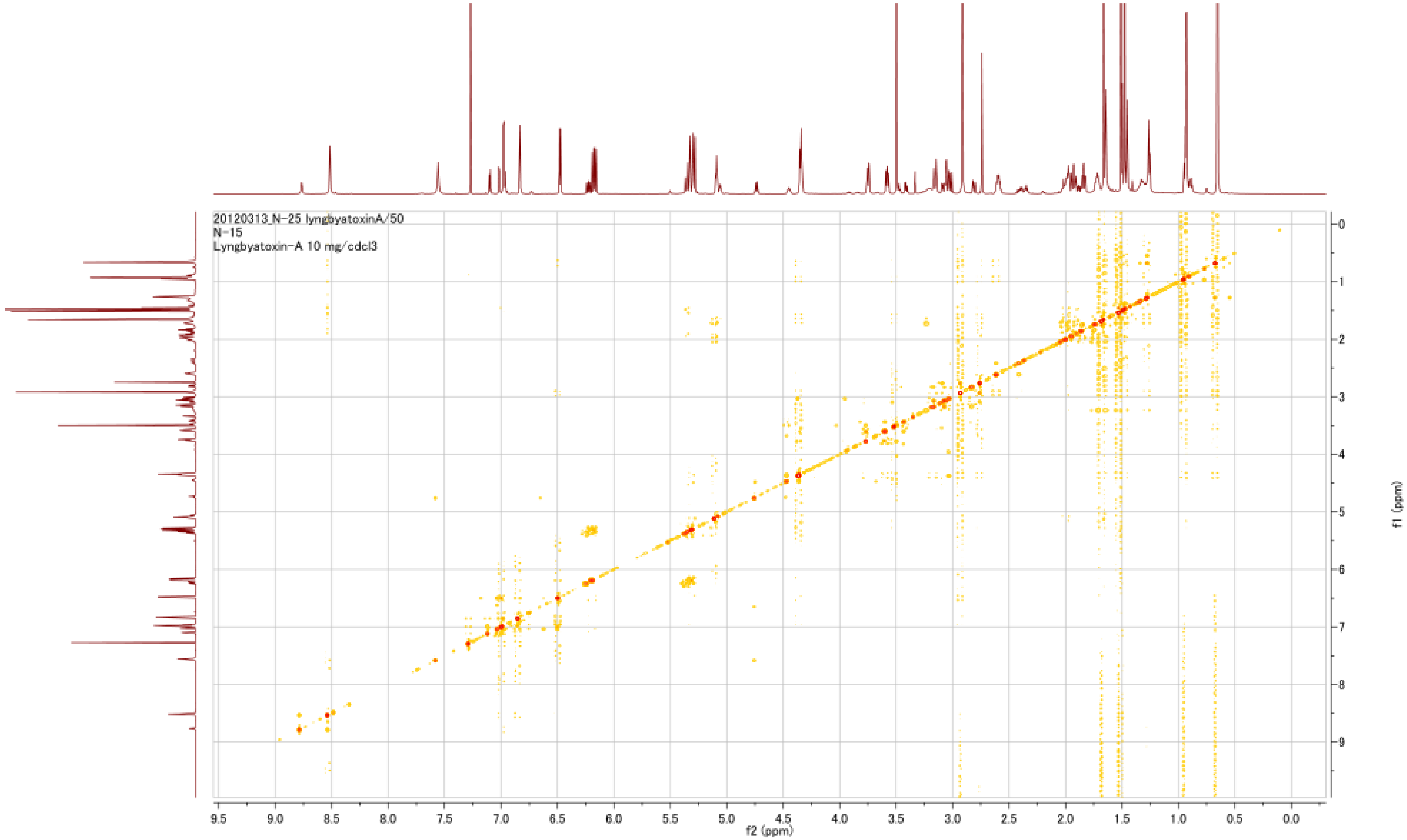

Figure S18. DEPT 45 spectrum of compound **2**, in $CDCl_3$.

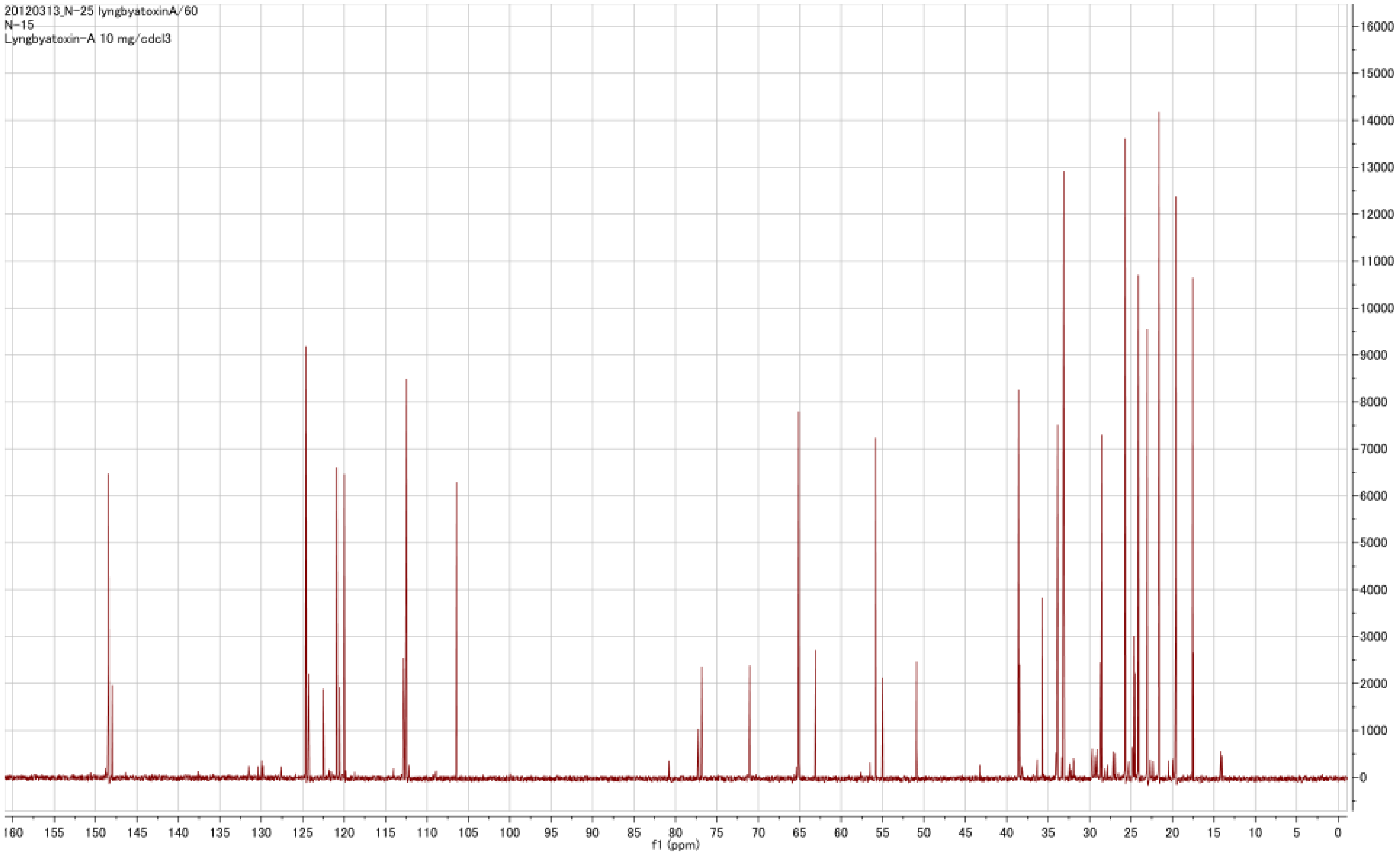

Figure S19. DEPT 90 spectrum of compound **2**, in $CDCl_3$.

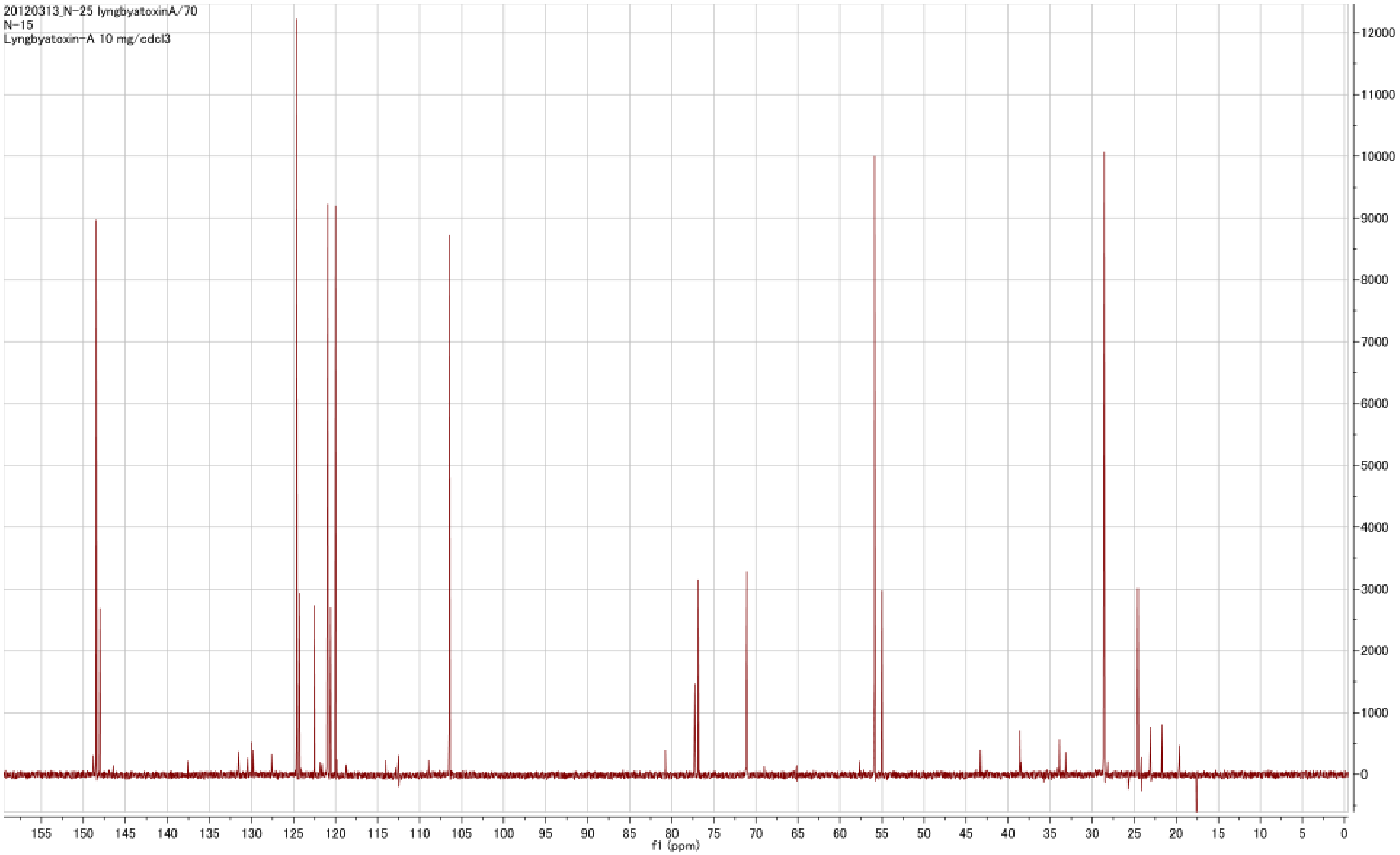

Figure S20. DEPT 135 spectrum of compound **2**, in $CDCl_3$.

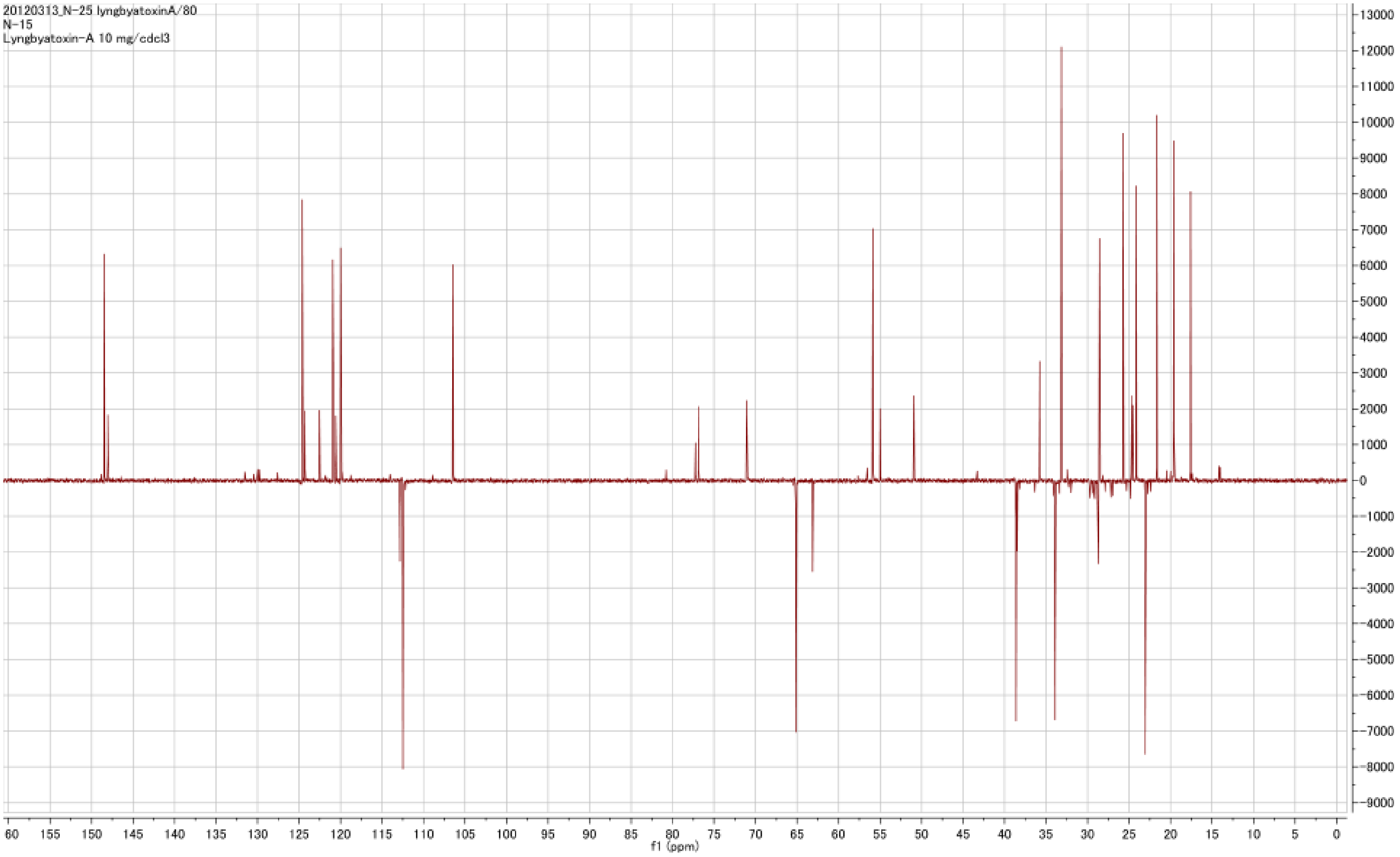

Figure S21. UV spectrum of compound **2**.

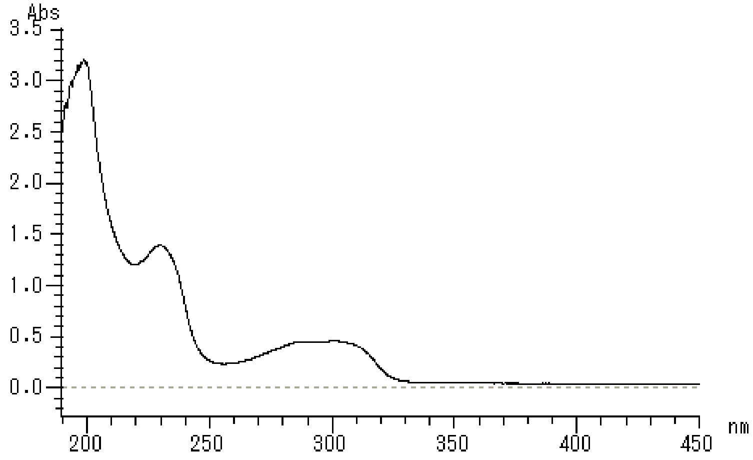

Figure S22. CD spectrum of compounds **1** [a], **2** [b], **3** [b], **4** [b] and **5** [b].

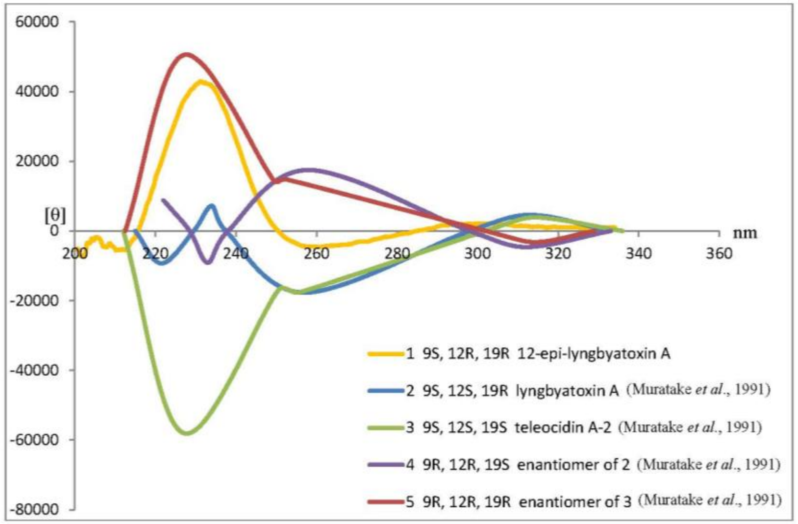

[a] The CD spectrum of compound **1** were achieved in our study; [b] The curves of CD spectrum are based on the reference [1] and then processed by Microsoft Excel.

Figure S23. ^{1}H-NMR spectrum about conformational ratio of compound **1**, in $CDCl_3$.

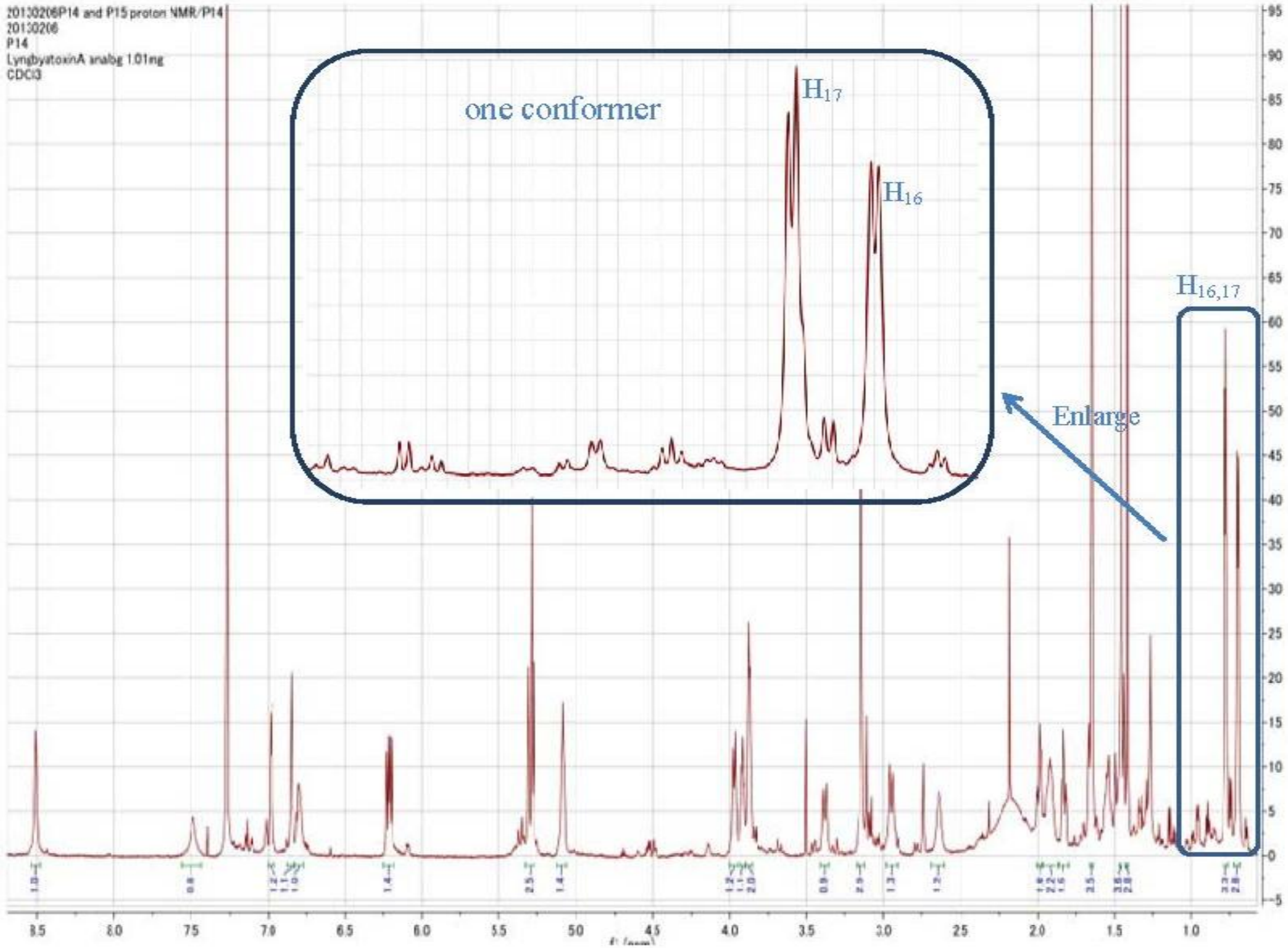

Figure S24. ^{1}H-NMR spectrum about conformational ratio of compound **2**, in $CDCl_3$.

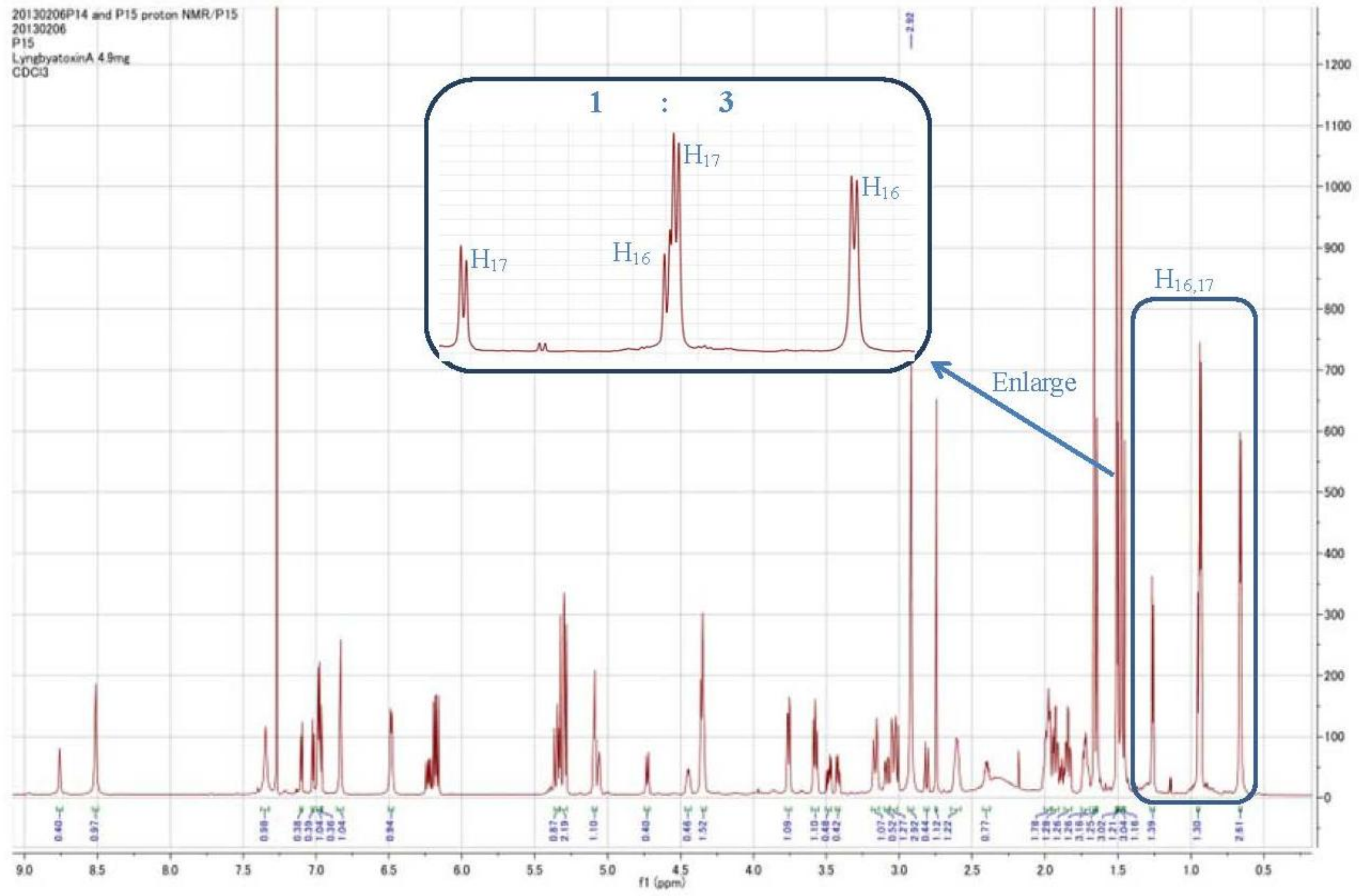

Figure S25. ^{1}H-NMR spectrum (600 MHz) of compound **1**, in CD_3OD.

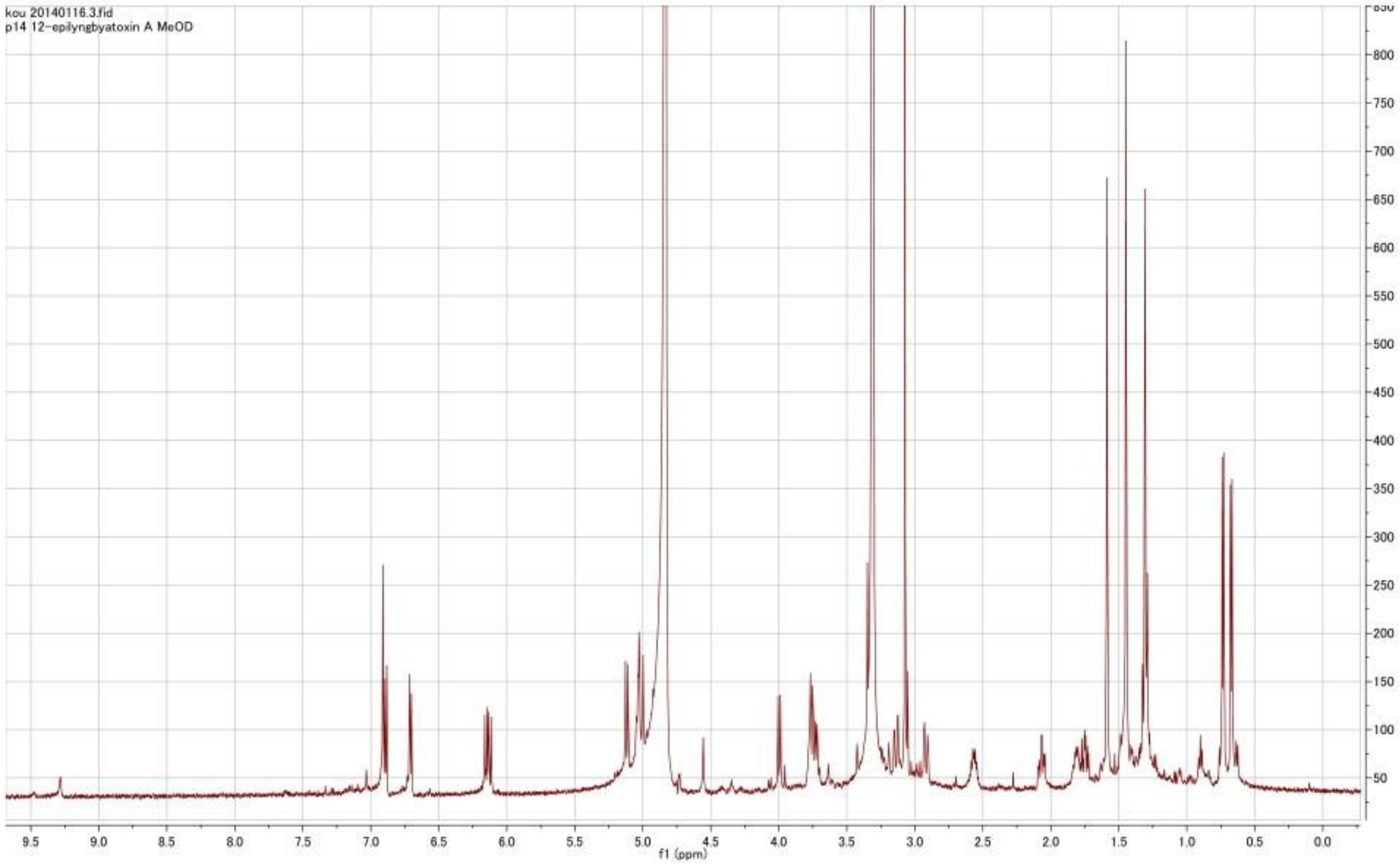

Figure S26. Optical rotations of IL-Vs and their related compounds (**1**).

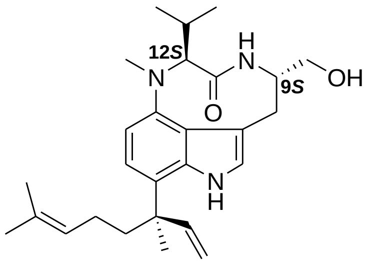

$[\alpha]^{D}_{25}$ = −171 °(*c* 1.8, $CHCl_3$) (3)

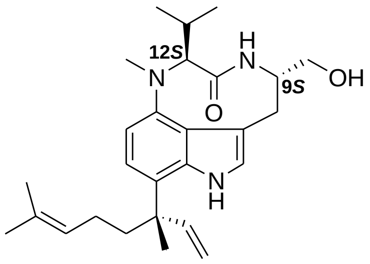

$[\alpha]^{D}_{18}$ = −185.1 °(*c* 0.18, CH_3OH) (18)

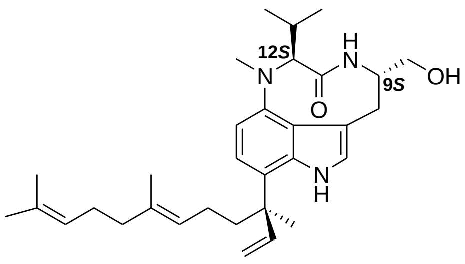

$[\alpha]^{D}_{24}$ = −147.2 °(*c* 0.67, $CHCl_3$) (20)

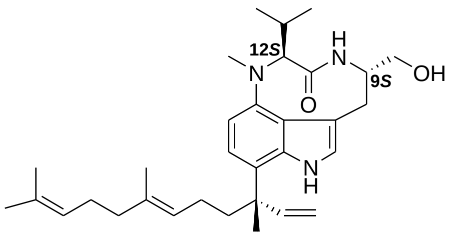

$[\alpha]^{D}_{24}$ = −206.4 °(*c* 0.49, $CHCl_3$)

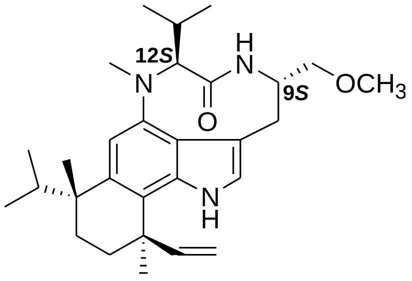

$[\alpha]^{D}$ = −314.9 °(in $CHCl_3$) (25)

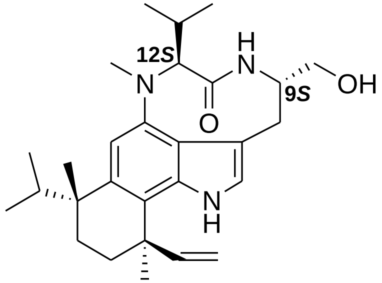

$[\alpha]^{D}$ = −141.5 °(in CH_3OH) (25)

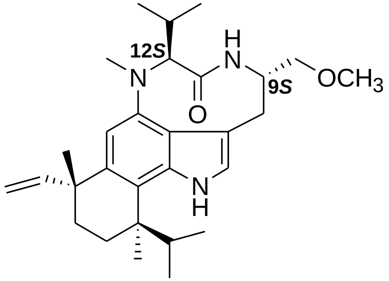

$[\alpha]^{D}_{32}$ = −256.6 °(c 0.07, $CHCl_3$) (25, 27)

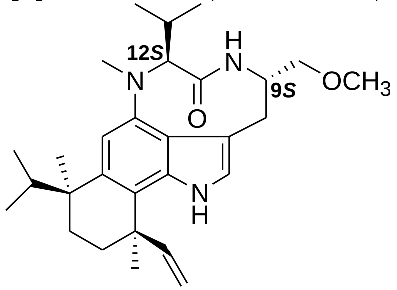

$[\alpha]^{D}_{32}$ = −298.4 °(c 0.35, $CHCl_3$) (25, 27)

The numbers in parentheses corresponded to the numbers of references in the original paper.

Figure S27. Optical rotations of IL-Vs and their related compounds (**2**).

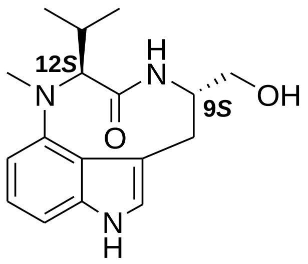

$[\alpha]^{D}_{20} = -134.8$ °(c 0.7, CH_3OH) (26)
$[\alpha]^{D}_{20} = -134.5$ °(c 0.7, CH_3OH) (28)

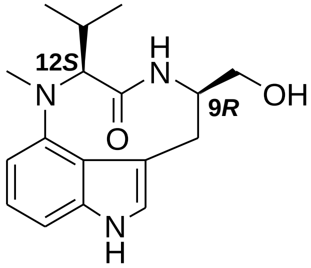

$[\alpha]^{D}_{20} = -84.9$ °
(c 0.75, CH_3OH) (26, 28)

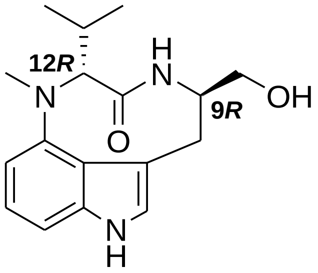

$[\alpha]^{D}_{20} = +135.6$ °(c 0.56, CH_3OH) (26)
$[\alpha]^{D}_{20} = +136.5$ °(c 0.56, CH_3OH) (28)

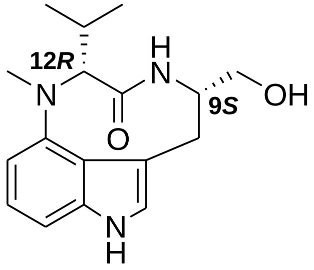

$[\alpha]^{D}_{20} = +86.5$ °
(c 0.83, CH_3OH) (26, 28)

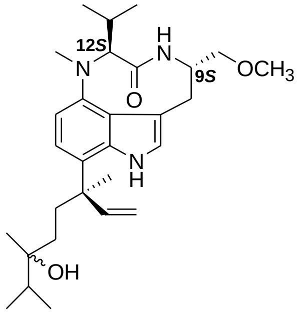

$[\alpha]^{D}_{23} = -51$ °
(c 0.27, MeOH) (30)

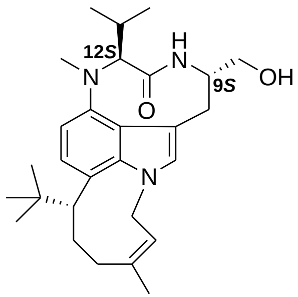

$[\alpha]^{D}_{22} = -64.7$ °
(c 0.63, EtOH) (30)

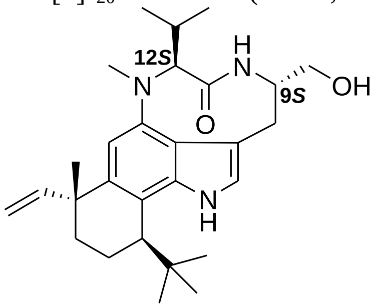

$[\alpha]^{D}_{24} = -112$ °
(c 0.25, EtOH) (30)

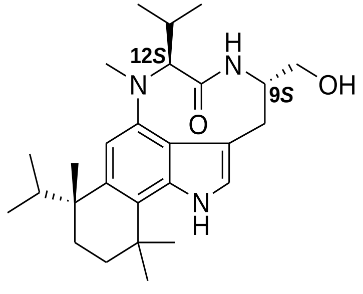

$[\alpha]^{D}_{32} = -353.8$ °
(c 0.5, $CHCl_3$) (33)

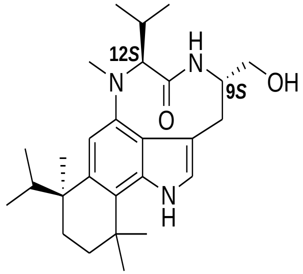

$[\alpha]^{D}_{25} = -153.8$ °
(c 0.5, $CHCl_3$) (33)

The numbers in parentheses corresponded to the numbers of references in the original paper.

Figure S28. Isolation and purification scheme of 12-*epi*-lyngbyatoxin A from the cyanobacterium.

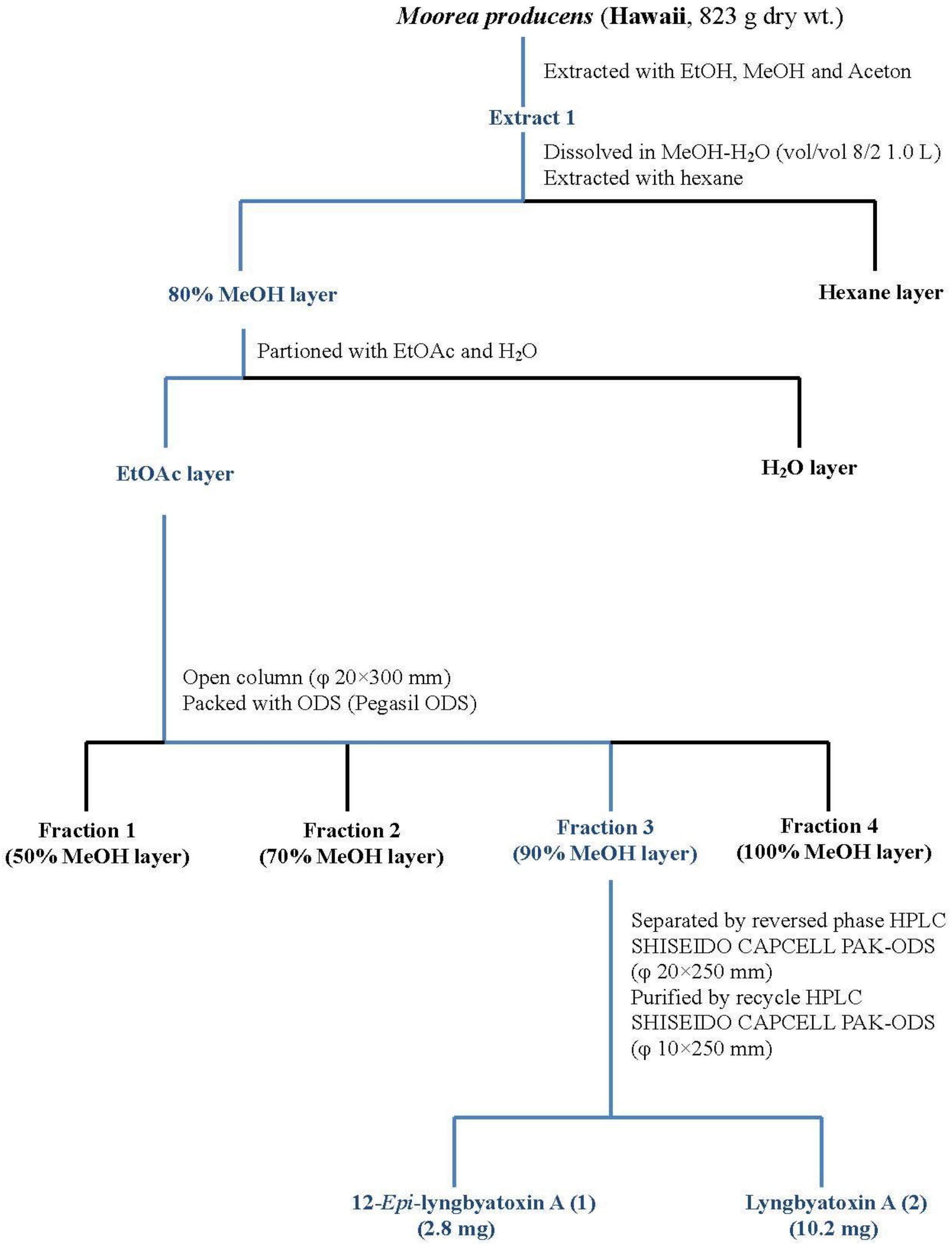

Figure S29. HPLC chromatogram for analysis of compound **1** (above) and **2** (below).

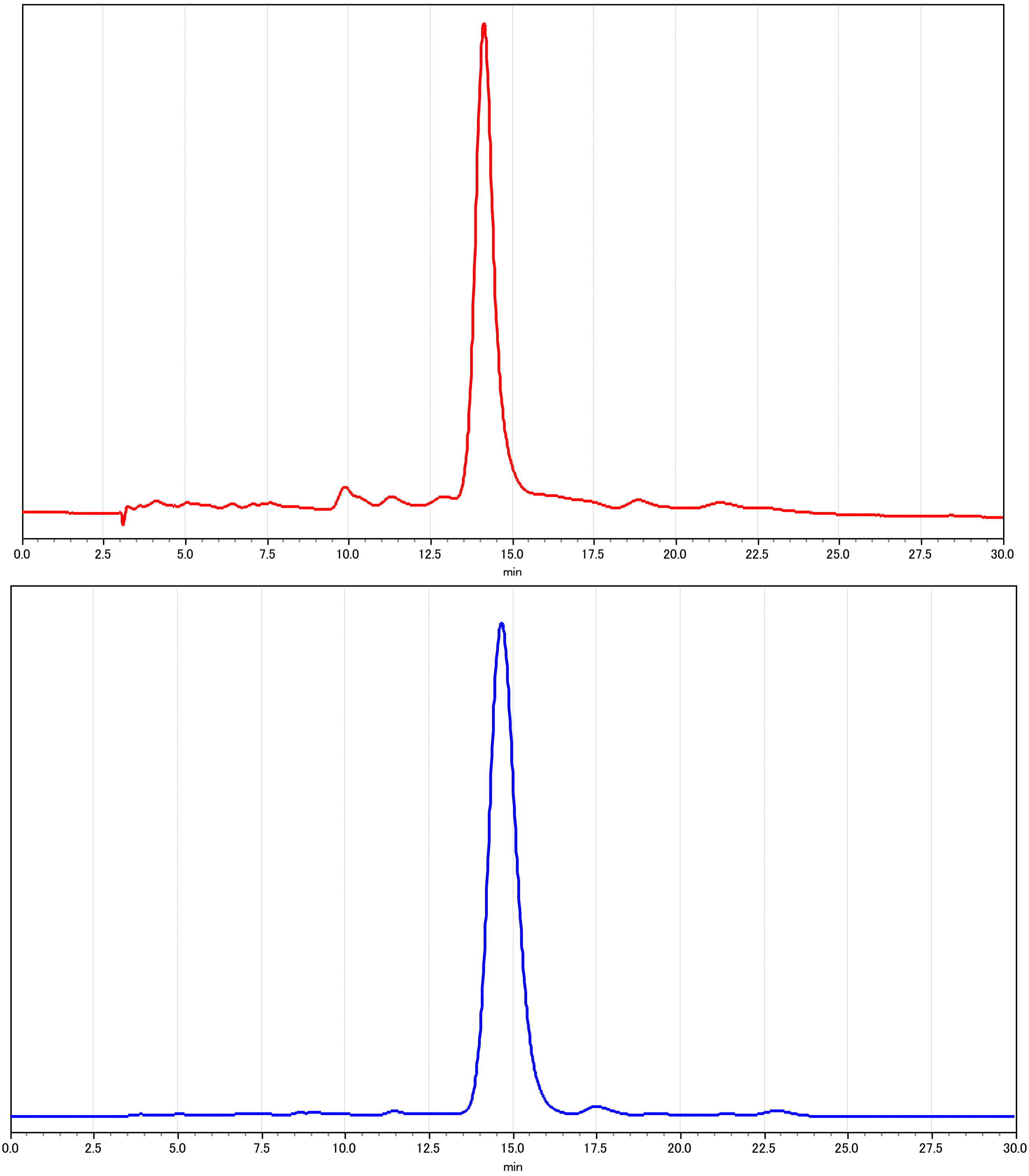

Column: SHISEIDOU CAPCELL PAK C18 Φ 4.6 mm × 250 mm; Isocratic solvent: 85% MeOH; Flow rate: 0.8 mL/min; Absorption: 260 nm; Injection sample: 10 μg/10 μL.

Table S1. ^{1}H and ^{13}C-NMR data for conformers *cis* and *trans* of compound **2** [a].

	***Cis* conformer**		***Trans* conformer**	
position	δC [b]	δH (*J* in Hz) [c]	δC [b]	δH (*J* in Hz) [c]
2	121.0	6.82, 1H, br s	124.4	6.95, 1H, d, *J* = 2.3 Hz
3	114.2		ND	
3a	118.9		ND	
4	146.5		ND	
5	106.6	6.47, 1H, d, *J* = 8.1 Hz	122.6	7.01, 1H, d, *J* = 7.8 Hz
6	120.1	6.97, 1H, d, *J* = 8.1 Hz	120.7	7.09, 1H, d, *J* = 7.8 Hz
7	121.7		ND	
7a	137.7		ND	
8	34.0	3.15, 1H, dd, *J* = 17.4, 3.7 Hz	28.8	3.08, 1H, dd, *J* = 14.8, 1.5 Hz
		3.04, 1H, dd, *J* = 17.4, 3.7 Hz		2.80, 1H, dd, *J* = 14.8, 1.5 Hz
9	56.0	4.33, 1H, br s	55.1	4.45, 1H, br m
11	174.8		ND	
12	71.2	4.34, 1H, d, *J* = 10.1 Hz	ND	
14	65.3	3.74, 1H, dd, *J* = 11.6, 3.5 Hz	63.3	3.47, 1H, dd, *J* = 11.2, 6.4 Hz
		3.57, 1H, dd, *J* = 11.6, 8.5 Hz		3.40, 1H, dd, *J* = 11.2, 7.3 Hz
15	28.7	2.59, 1H, m	24.7	2.38, 1H, br m
16	19.7	0.64, 3H, d, *J* = 6.8 Hz	19.8	0.93, 3H, d, *J* = 6.5 Hz
17	21.8	0.92, 3H, d, *J* = 6.3 Hz	19.7	1.25, 3H, d, *J* = 6.6 Hz
18	33.3	2.90, 3H, s	35.9	2.74, 3H, s
19	43.4		ND	
20	25.8	1.65, 3H, s	25.8	1.63, 3H, s
21	148.6	6.16, 1H, dd, *J* = 17.8, 10.7 Hz	148.1	6.22, 1H, dd, *J* = 17.7, 10.7 Hz
22	112.6	5.30, 1H, dd, *J* = 17.8, 1.2 Hz	113.0	5.35, 1H, dd, *J* = 17.8, 1.1 Hz
		5.28, 1H, dd, *J* = 10.8, 1.2 Hz		5.33, 1H, dd, *J* = 10.8, 1.1 Hz
23	38.7	1.92, 1H, td, *J* = 12.7, 12.7, 4.1 Hz	ND	
		1.83, 1H, td, *J* = 12.7, 12.7, 4.1 Hz		
24	23.2	1.97, 1H, br m	ND	
		1.71, 1H, br m		
25	124.7	5.08, 1H, br m	124.4	5.05, 1H, br m
26	131.7		ND	
27	24.3	1.47, 3H, s	24.8	1.49, 3H, s
28	17.7	1.50, 3H, s	17.6	1.44, 3H, s
OH on 14		Not observed		

[a] All data were recorded in $CDCl_3$; ^{1}H-^{13}C connectivities assigned by HSQC experiment; [b] Recorded at 200 MHz; [c] Recorded at 800 MHz. Coupling constants (Hz) are in parentheses; Abbreviations: s, singlet; d, doublet; t, triplet; m, multiplet; br, broad; ND: Not unambiguously Determined.

Table S2. ^{1}H-NMR data of (+)-*epi*-indolactam V and compound **1**.

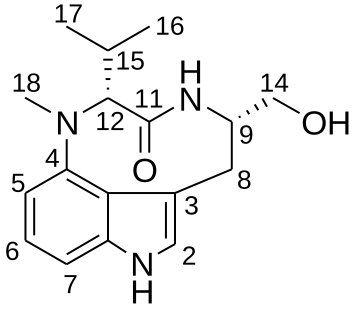

(+)-*Epi*-indolactam V

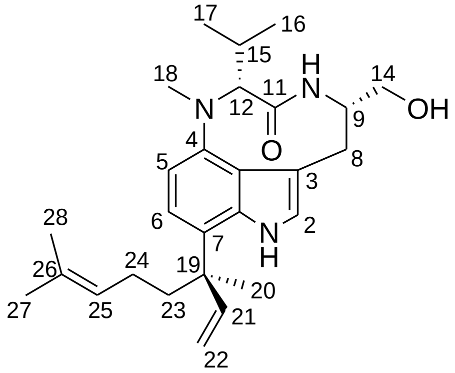

compound **1** (12-*Epi*-lyngbyatoxin A)

(+)-*Epi*-indolactam V		Compound 1	
proton	δH (*J* in Hz) [a,b]	proton	δH (*J* in Hz) [a,c]
H2	6.90 (s)	H2	6.91 (s)
H5	6.66	H5	6.71 (d, *J* = 7.9 Hz)
H6	6.92	H6	6.89 (d, *J* = 7.9 Hz)
H7	6.91		
H8	2.94 (dd, *J* = 15.1, 2.2 Hz)	H8	2.92 (dd, *J* = 15.3, 2.5 Hz)
	3.10 (dd, *J* = 15.1, 3.7 Hz)		3.14 (dd, *J* = 15.3, 2.7 Hz)
H9	3.80 (m)	H9	3.77 (m)
H12	4.03 (d, *J* = 10.5 Hz)	H12	4.00 (d, *J* = 10.7 Hz)
H14	3.72 (dd, *J* = 11.5, 8.1 Hz)	H14	3.72 (dd, *J* = 11.4, 3.5 Hz)
	3.77 (dd, *J* = 11.5, 5.8 Hz)		3.76 (dd, *J* = 11.4, 4.8 Hz)
H15	2.57 (dsept, *J* = 10.5, 6.9 Hz)	H15	2.56 (m)
H16,17	0.69 (d, *J* = 6.9 Hz)	H16,17	0.67 (d, *J* = 6.6 Hz)
	0.75 (d, *J* = 6.9 Hz)		0.73 (d, *J* = 6.6 Hz)
H18	3.08 (s)	H18	3.07 (s)

[a] All data were recorded in MeOD; [b] These ^{1}H-NMR data are achieved from the reference [2]; [c] Recorded at 600 MHz. Coupling constants (Hz) are in parentheses.

References

1. Muratake, H.; Okabe, K.; Natsume, M. Synthesis of teleocidins A, B and their congeners. Part 2. Synthesis of lyngbyatoxin A (teleocidin A-1), teleocidin A-2, pendolmycin, and (*R*,*E*)- and (*S*,*E*)-7-(3,7,11-trimethyl-1,6,10-dodecatrien-3-yl)-(−)-indolactams V. *Tetrahedron* **1991**, *47*, 8545–8558.
2. Endo, Y.; Shudo, K.; Itai, A.; Hasegawa, M.; Sakai, S.I. Synthesis and stereochemistry of indolactam-V, an active fragment of teleocidins. Structural requirements for tumor-promoting activity. *Tetrahedron* **1986**, *42*, 5905–5924.

© 2014 by the authors; licensee MDPI, Basel, Switzerland. This article is an open access article distributed under the terms and conditions of the Creative Commons Attribution license (http://creativecommons.org/licenses/by/3.0/).
